# Evaluation of Copathology and Clinical Trajectories in Individuals With Tau-Clinical Mismatch

**DOI:** 10.1001/jamaneurol.2025.4974

**Published:** 2025-12-15

**Authors:** Christopher A. Brown, Nidhi S. Mundada, Katheryn A. Q. Cousins, Niyousha Sadeghpour, Xueying Lyu, Emily McGrew, Magdalena Korecka, Alice Chen-Plotkin, Long Xie, Laura E. M. Wisse, John A. Detre, Corey T. McMillan, Edward B. Lee, Ilya M. Nasrallah, Sandhitsu R. Das, Dawn Mechanic-Hamilton, Paul A. Yushkevich, Leslie M. Shaw, David A. Wolk

**Affiliations:** 1 Department of Neurology, University of Pennsylvania, Philadelphia; 2 Department of Bioengineering, University of Pennsylvania, Philadelphia; 3 Department of Radiology, University of Pennsylvania, Philadelphia; 4 Department of Pathology and Laboratory Medicine, University of Pennsylvania, Philadelphia; 5 Department of Digital Technology and Innovation, Siemens Healthineers, Princeton, New Jersey; 6 Department of Clinical Sciences, Lund University, Lund, Sweden

## Abstract

**Importance:**

Even within individuals who are amyloid positive, clinical symptoms can be impacted by Alzheimer pathology, other pathologic proteins, and cognitive reserve or resilience. Understanding how each of these factors contributes to a patient’s clinical presentation is crucial for prognosis and treatment decisions in the era of disease-modifying therapies.

**Objective:**

To evaluate tau-clinical mismatch as a means to identify those more likely to harbor copathology and/or exhibit resilience to Alzheimer pathology.

**Design, Setting, and Participants:**

This was a longitudinal, observational cohort study conducted from 2004 to 2024. The setting included multiple academic medical centers in the US participating in the Alzheimer’s Disease Neuroimaging Initiative (ADNI) or Penn Alzheimer’s Disease Research Center (Penn-ADRC). Included in the analysis were individuals with clinical assessment and measures of either tau positron emission tomography (tau-PET) or phosphorylated tau 217 (p-tau217) who were selected from individuals positive for amyloid β (Aβ+) in the ADNI cohort (aged 55-95 years) and the Penn-ADRC cohort (aged 54-92 years).

**Exposures:**

Clinical assessment (Clinical Dementia Rating Sum of Boxes [CDR-SB]) and tau burden (tau-PET or p-tau217) for mismatch group classification.

**Main Outcomes and Measures:**

Cross-sectional measures of neurodegeneration (medial temporal lobe volume and thickness, cortical thickness, TAR DNA-binding protein 43 [TDP-43] imaging signature), α-synuclein cerebrospinal fluid seed-amplification assay, and longitudinal CDR-SB.

**Results:**

A total of 365 participants (mean [SD] age, 75.4 [7.9] years; 192 female [52.6%]) in the ADNI tau-PET group and 524 participants (mean [SD] age, 77.1 [7.9] years; 268 male [51.1%]) in the ADNI p-tau217 group were selected from the 998 individuals who were Aβ+ in the ADNI cohort and used to generate tau-clinical mismatch models with 55.6% to 57.1% classified as canonical (CDR-SB commensurate to tau), 23.7% to 24.7% as resilient (CDR-SB < tau), and 19.3% to 19.7% as vulnerable (CDR-SB > tau). Vulnerable groups had evidence of greater likelihood of copathology, with TDP-43 neurodegeneration patterns and α-synuclein positivity. Mismatch groups showed diverging clinical trajectories with earlier cognitive impairment in vulnerable groups and later impairment in resilient individuals. Similar findings were seen when applied to an independent dataset of 244 individuals (mean [SD] age, 73.7 [6.8] years; 139 female [57.0%]) of the 248 who were Aβ+ in Penn-ADRC cohort. Finally, these models were applied to a cohort receiving antiamyloid therapy to show the utility of this method for predicting individual cognitive trajectories during therapy.

**Conclusion and Relevance:**

Results of this cohort study suggest that tau-clinical mismatch identified individuals more likely to have an accelerated disease course due to the presence of copathology and those exhibiting greater cognitive resilience to disease pathology. These models provide an important tool that can be implemented in clinical practice to provide improved individualized prognosis and, potentially, monitoring of response to disease-modifying therapy.

## Key Points

**Question:**

Does tau-clinical mismatch identify individuals with copathology and differing clinical trajectories?

**Findings:**

In the Alzheimer’s Disease Neuroimaging Initiative and Penn Alzheimer’s Disease Research Center cohorts including a total of 878 participants, those with greater clinical impairment than expected for tau burden had evidence of significantly higher rates of copathology and faster clinical decline compared with those with the expected level of impairment for a given level of tau pathology.

**Meaning:**

Results suggest that when individuals have a higher level of clinical impairment than expected for tau, they are more likely to have copathology and are likely to experience faster decline, which may impact treatment decisions and evaluation of response.

## Teaser

This cohort study investigates if tau-clinical mismatch identifies individuals with copathology and differing clinical trajectories.

## Introduction

With the approval of amyloid-targeting therapies (ATTs) for Alzheimer disease (AD), biomarker testing for amyloid β (Aβ) status has dramatically increased in clinical practice. Importantly, Aβ itself does not cause significant clinical impairment, which instead is associated with downstream accumulation of tau pathology.^1,2,3^ Moreover, non-AD copathologies, such as TAR DNA-binding protein 43 (TDP-43) and α-synuclein, as well as factors such as cognitive reserve and resilience all contribute to an individual’s clinical presentation.^4,5^ This makes it challenging for clinicians to assess the degree to which the clinical impairment of an individual who is Aβ+ is due to AD vs these other factors, complicating both prognostic guidance and treatment decisions. Therefore, there is a strong need for clinical tools to address the degree to which each factor may be contributing to clinical impairment in an individual patient.

The 2024 Alzheimer Association workgroup guidelines for the diagnosis and staging of AD attempt to address this issue by providing a framework that first asks clinicians to separately stage individuals biologically based on tau burden and clinically based on their level of impairment.^6^ Once individuals are staged in each dimension, the clinical and biological stage can be compared for congruence (termed *canonical*) or mismatch with either biological stage greater than clinical stage (termed *resilient*) or clinical stage greater than biological stage (termed *vulnerable*). Within this framework, individuals classified as canonical have symptoms primarily attributable to underlying AD pathology, those who are vulnerable may have copathology or increased susceptibility to AD, and those who are resilient likely have cognitive reserve or resilience.^6^ Recent efforts to operationalize these criteria have shown their potential benefit by demonstrating that vulnerable individuals exhibit greater evidence of copathology.^7^

However, there are 2 primary barriers to implementing these criteria into clinical practice: (1) the use of discrete stages and cutoffs prevents empiric evaluation of the normative canonical tau-clinical association, and (2) tau stages are defined based on tau positron emission tomography (tau-PET), which has limited clinical availability and is resource intensive. Here, we aim to address both limitations by using continuous measures of tau burden and clinical impairment to empirically investigate the tau-clinical association and define mismatch using a widely available blood-based biomarker, phosphorylated tau 217 (p-tau217) vs tau-PET as measures of tau burden. Although often considered a biomarker of Aβ pathology due to its strong discrimination between Aβ-positive (Aβ+) or Aβ-negative (Aβ−) individuals, many studies have now demonstrated the stronger association of p-tau217 with tau burden compared with amyloid burden, particularly within Aβ+ individuals.^8,9,10,11^ After developing these tau-clinical mismatch models, we investigated differences in copathology and longitudinal clinical trajectories between groups before replicating our results in an independent dataset and illustrating their potential utility in practice in the setting of ATTs.

## Methods

This study was approved by the University of Pennsylvania institutional review board (IRB) and local IRBs at participating Alzheimer’s Disease Neuroimaging Initiative (ADNI) sites. All participants provided written informed consent as detailed in the ADNI protocol. All participants in the Penn Alzheimer’s Disease Research Center (Penn-ADRC) cohort provided written informed consent under protocols approved by the University of Pennsylvania institutional review board. This study followed the Strengthening the Reporting of Observational Studies in Epidemiology (STROBE) reporting guidelines.

### Participants

Individuals were selected from the ADNI dataset based on the following: (1) positive Aβ PET, (2) at least 1 tau-PET or Fujirebio Lumipulse p-tau217 measure, and (3) at least 1 Clinical Dementia Rating Sum of Boxes (CDR-SB) score. All participants also had a magnetic resonance imaging (MRI) scan available, and a subset had available cerebrospinal fluid α-synuclein seed amplification assay (SAA) and longitudinal clinical data. A replication dataset was selected from the Penn-ADRC clinical care cohort based on (1) Fujirebio Lumipulse p-tau217/Aβ42 ratio greater than 0.0055 (Aβ+ cutoff)^12^ and (2) at least 1 CDR-SB score. A subset of these participants also had available MRI and longitudinal clinical data. Finally, to illustrate clinical application, a dataset of individuals receiving ATT at the Penn Memory Center and participating in an ongoing observational study (the Penn-ATM study) were selected based on availability of the following: (1) Fujirebio Lumipulse p-tau217, (2) Mini-Mental State Examination (MMSE), and (3) Dementia Severity Rating Scale (DSRS).^13^ Participants in all studies provided self-reported race (Asian, Black, multiracial, and White), which was included for the purpose of better characterizing the study population.

### Assessment of Tau Pathology

^18^F-flortaucipir tau-PET acquisition and plasma collection were performed as previously described (eMethods in Supplement 1).^8^ For tau-PET, we calculated global Tau-MaX, which provides a region-agnostic assessment of global tau magnitude and extent with values ranging from 0 to 100.^8^ Fujirebio Lumipulse p-tau217 was used as a measure of tau burden in the ADNI-plasma, Penn-ADRC, and Penn-ATM datasets.

### Clinical Assessment

CDR-SB was selected as the primary measure of clinical impairment due its blend of functional and cognitive impairment, as well as its use as a primary clinical end point in phase 3 trials of ATT.^14,15^ For both the ADNI and Penn-ADRC cohorts, the CDR-CB value obtained closest in time to tau-PET or p-tau217 collection was used for cross-sectional analyses. In addition, all available measures of CDR-SB and all available MMSE (ADNI) or Montreal Cognitive Assessment (Penn-ADRC) scores were used for longitudinal analyses. Clinical diagnosis determined through consensus procedures previously detailed for ADNI^16^ and Penn-ADRC^17^ were used to assess progression to the next stage of clinical impairment. For the Penn-ATM study, the most recent MMSE and DSRS scores before starting ATT was used for analyses.

### Assessment of Neurodegeneration and Copathology Signatures

T1-weighted MRI was used to assess multiple structural parameters (eMethods in Supplement 1). In addition to whole-brain analyses, we focused on more granular measures of medial temporal lobe (MTL) structure due its involvement in TDP-43 pathology.^18,19^ Further, we calculated a TDP-43-associated MRI signature consisting of the ratio of entorhinal cortex (ERC) thickness to parahippocampal cortex (PHC) thickness, which is reduced in individuals with autopsy-proven TDP-43 positivity.^20^ Finally, we evaluated α-synuclein SAA in the cerebrospinal fluid as a measure of α-synuclein copathology in ADNI^21^ (eMethods in Supplement 1).

### Statistical Analyses

Continuous variables are presented as mean (SD), and categorical variables are presented as number and percentage. All *P* values were 2-sided, and *P* <.05 was considered statistically significant. All analyses were performed in R, version 4.4.1 (R Project for Statistical Computing) with additional visualizations using ParaView (Kitware Inc) and Surf Ice (Chris Rorden^22^). Outliers identified as greater than 5 SDs from the mean were excluded.

To determine the empiric tau-clinical association and define mismatch, we first examined the association between tau burden (Tau-MaX or p-tau217) and clinical impairment (CDR-SB) using linear regression while controlling for age, sex, and education in the ADNI datasets. Standardized residuals (SR) were extracted for each participant to classify individuals with low residual (−0.6≤ SR ≤0.6) as canonical, those with highly positive residual (SR >0.6) representing greater cognitive impairment than expected for tau as vulnerable, and those with highly negative residual (SR <−0.6) indicating less clinical impairment than expected based for as resilient. Although this cutoff is based on prior work examining tau-neurodegeneration mismatch,^23^ sensitivity analyses were performed for all cross-sectional analyses using the mismatch SR as a continuous measure.

After defining tau-clinical mismatch, we next evaluated cross-sectional differences in markers of copathology (TDP-43 MRI signature and α-synuclein SAA) and brain structure between groups. For each analysis, age, sex, education, and tau burden (Tau-MaX or p-tau217) were included as covariates, and the tau-clinical mismatch group was the predictor of interest. Linear regression was used for continuous outcomes, and logistic regression was used for dichotomous outcomes. Additional details including correction for multiple comparisons are provided in the eMethods in Supplement 1.

We then sought to evaluate whether tau-clinical mismatch identified individuals on differing longitudinal trajectories. Before longitudinal analyses, we used sampled iterative local approximation^24^ to first place individuals onto a common biological timeline anchored to estimated tau onset age (ETOA) as detailed in the eMethods in Supplement 1. Thus, time for longitudinal analysis was time from ETOA rather than an arbitrary time from the collection of tau-PET or p-tau217. We then used linear mixed models with either CDR-SB or MMSE as the outcome variable, tau-clinical mismatch group × time from ETOA (with a b-spline with 3 degrees of freedom) as the interaction of interest, age, sex, education, and baseline tau burden (Tau-MaX or p-tau217) as covariates, and a random effect of participant. Finally, we examined differences between mismatch groups in time to progress to the next clinical stage using Cox-proportional hazards models.

After developing these models in ADNI, we applied the ADNI p-tau217 model to the Penn-ADRC dataset and compared this applied classification to repeating model development within Penn-ADRC. We then examined differences in TDP-43 MRI signature, brain structure, and longitudinal cognitive progression between mismatch groups as described previously in the ADNI dataset. Finally, we applied the ADNI p-tau217 model to baseline data from the Penn-ATM dataset to demonstrate its potential clinical utility.

## Results

### Determining the Empiric Canonical Tau-Clinical Association and Defining Tau-Clinical Mismatch

**Figure 1.**
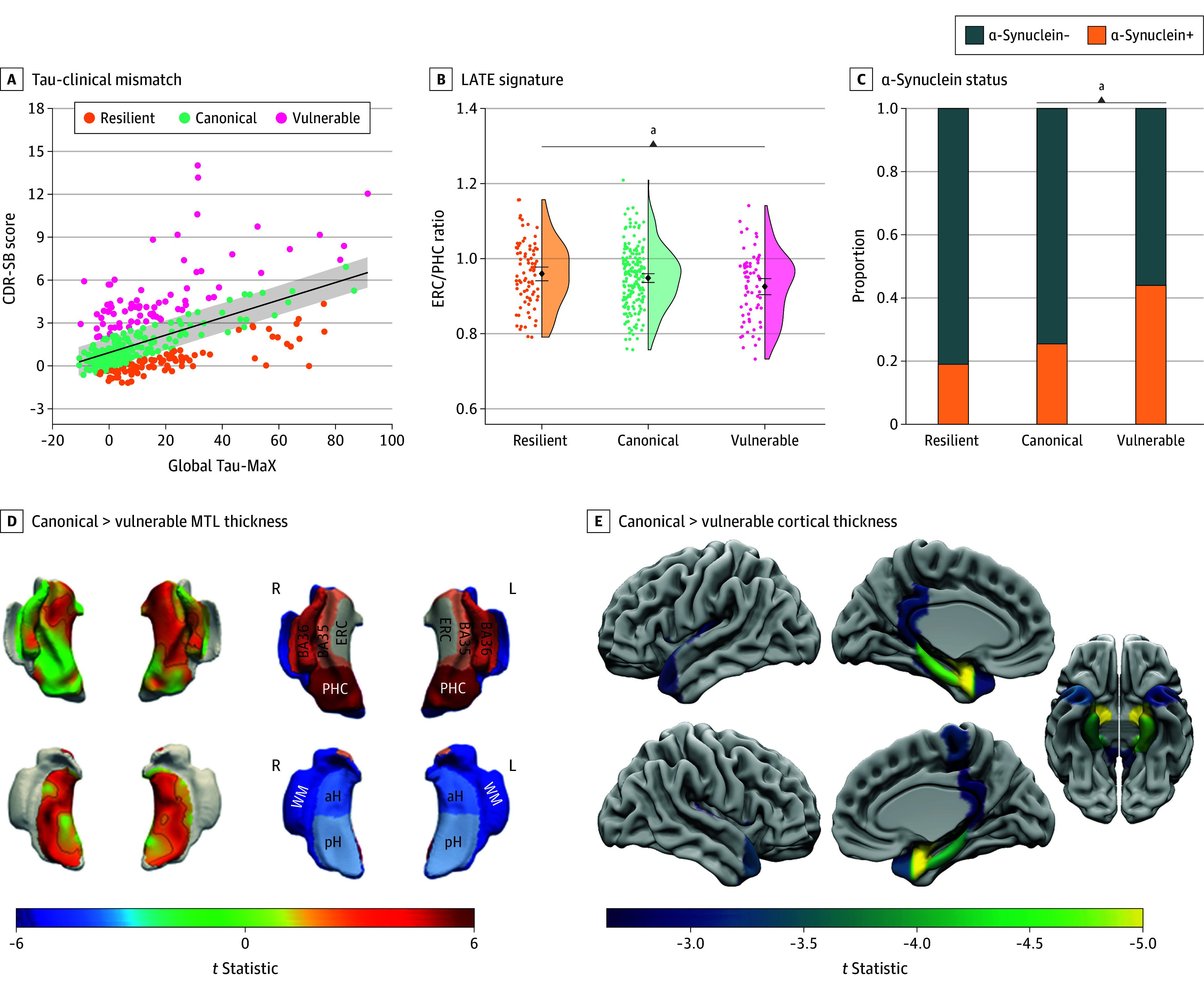
Cross-Sectional Differences in Atrophy and Copathology Between Alzheimer’s Disease Neuroimaging Initiative (ADNI) Tau Positron Emission Tomography (Tau-PET) Mismatch Groups A, Association between Clinical Dementia Rating Sum of Boxes (CDR-SB) and region-agnostic assessment of global tau magnitude (Tau-MaX) with mismatch classification shown by color scale (β = 0.53; 95% CI, 0.44-0.61; *t* = 11.7; *P* < .001). Gray shading represents standardized residual (SR) = 0.6. Comparison of copathology levels between mismatch groups are shown with limbic-predominant age-related TDP-43 encephalopathy (LATE)–magnetic resonance imaging signature entorhinal cortex thickness/parahippocampal cortex thickness (ERC/PHC) ratio (B) and α-synuclein status (C). Differences in medial temporal lobe (MTL) thickness (D) and whole-brain region of interest (ROI) thickness (E) between vulnerable and canonical mismatch groups with color-scale representing *t* statistics for group comparison. For the MTL thickness analysis, clusters surviving threshold-free cluster enhancement (TFCE) familywise error *P* <.05 are outlined in black with vulnerable participants showing decreased thickness in anterior MTL regions along with posterior hippocampus. For whole-brain ROI analysis, only regions surviving false discovery rate *P *<.05 shown with vulnerable participants showing decreased thickness in primarily temporopolar regions. aH indicates anterior hippocampus; BA, Brodmann area; pH, posterior hippocampus; WM, white matter. ^a^*P* < .05.

**Figure 2.**
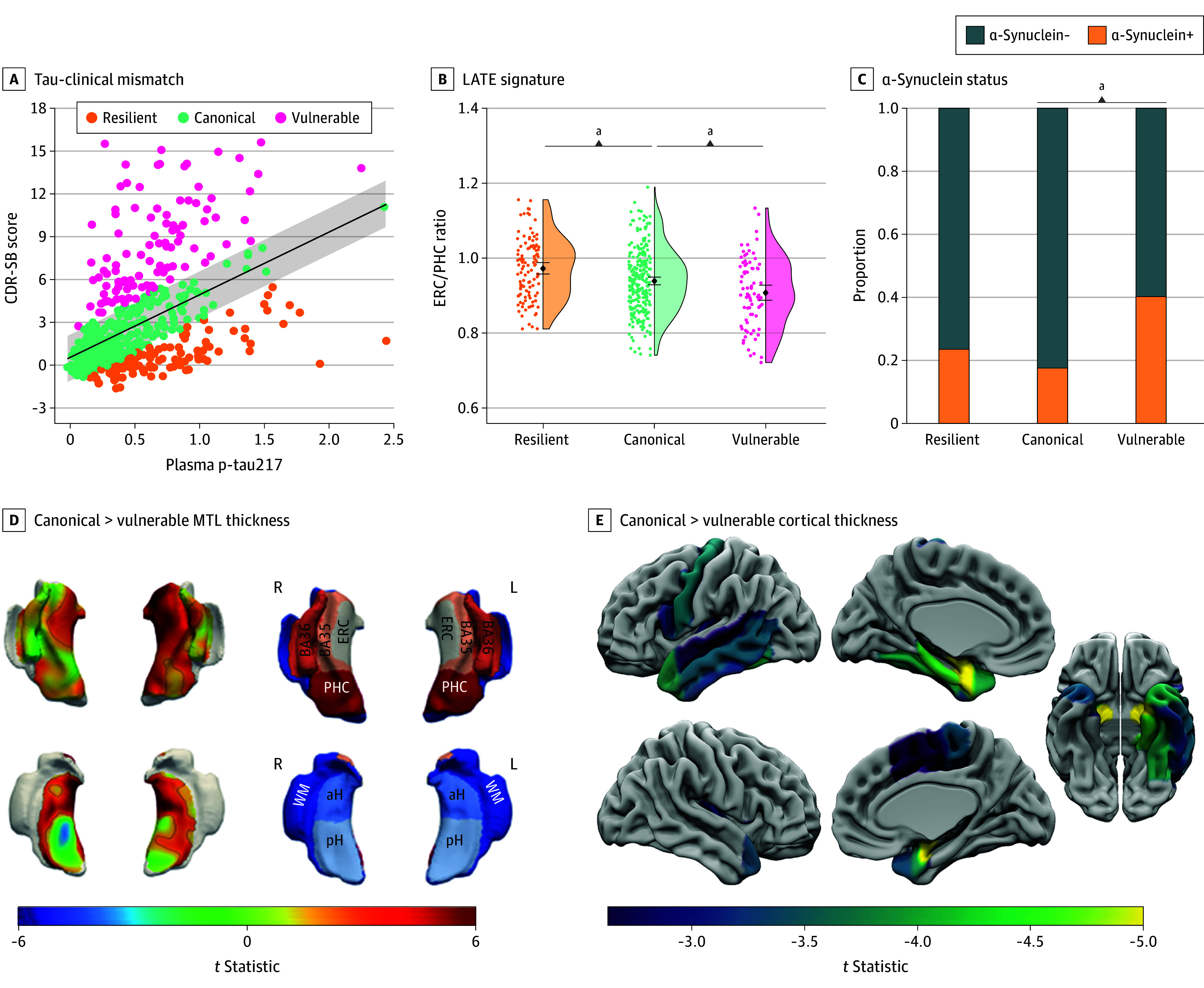
Cross-Sectional Differences in Atrophy and Copathology Between Alzheimer’s Disease Neuroimaging Initiative (ADNI) Phosphorylated Tau217 (p-Tau217) Mismatch Groups A, Association between Clinical Dementia Rating Sum of Boxes (CDR-SB) and p-tau217 with mismatch classification shown by color scale (β = 0.47; 95% CI, 0.39-0.54; *t* = 12.4; *P* < .001). Gray shading represents standardized residual (SR) = 0.6. Comparison of copathology levels between mismatch groups are shown with limbic-predominant age-related TDP-43 encephalopathy (LATE)–magnetic resonance imaging signature entorhinal cortex thickness/parahippocampal cortex thickness (ERC/PHC) ratio (B) and α-synuclein status (C). Differences in medial temporal lobe (MTL) thickness (D) and whole-brain region of interest (ROI) thickness (E) between vulnerable and canonical mismatch groups with color-scale representing *t *statistics for group comparison. For the MTL thickness analysis, clusters surviving threshold-free cluster enhancement (TFCE) familywise error *P *<.05 are outlined in black with vulnerable participants showing decreased thickness in anterior MTL regions along with posterior hippocampus. For whole-brain ROI analysis, only regions surviving false discovery rate *P *<.05 shown with vulnerable participants showing decreased thickness in primarily temporopolar regions. aH indicates anterior hippocampus; BA, Brodmann area; pH, posterior hippocampus; WM, white matter. ^a^*P* < .05.

A total of 365 Aβ+ individuals (mean [SD] age, 75.4 [7.9] years; 192 female [52.6%]; 173 male [47.4%]; 5 Asian [1.4%]; 32 Black [8.8%]; 9 multiracial [2.5%]; 319 White [87.3%]) in the ADNI tau-PET group and 524 Aβ+ individuals (mean [SD] age, 77.1 [7.9] years; 256 female [48.9%]; 268 male [51.1%]; 8 Asian [1.5%]; 35 Black [6.7%]; 11 multiracial [2.1%]; 470 White [89.7%]) in the ADNI plasma p-tau217 group were selected from the 998 individuals who were Aβ+ in the ADNI cohort and included in the analyses (eFigure 1 in Supplement 1). There was a strong positive association between Tau-MaX and CDR-SB (β = 0.53; 95% CI, 0.44-0.61; *t*_360_ = 11.7; *P* < .001) and using an SR ± 0.6 cutoff, 203 individuals (55.6%) were classified as canonical, 90 (24.7%) as resilient, and 72 (19.7%) as vulnerable (Figure 1A and eResults in Supplement 1). We then repeated this process using plasma p-tau217, which found a strong association between p-tau217 and CDR-SB (β = 0.47; 95% CI, 0.39-0.54; *t*_319_ = 12.4; *P* < .001) and resulted in 299 individuals (57.1%) classified as canonical, 124 (23.7%) classified as resilient, and 101 (19.3%) as vulnerable (Figure 2A).

A subset of 258 Aβ+ individuals had both tau-PET and p-tau217 available, and there was a strong positive association between these measures before and after controlling for Centiloids (eFigure 2 in Supplement 1). There was strong agreement between tau-clinical mismatch classification using either Tau-MaX or p-tau217 with 182 of 258 (71%) concordance and only 1 of 258 (0.4%) with vulnerable-resilient discordance (eTable 1 in Supplement 1).

### Cross-Sectional Differences Between Tau-Clinical Mismatch Groups

Demographic and summary measures for each mismatch group are shown in the Table. For both the tau-PET and p-tau217 models, canonical participants tended to be younger, female, and have lower amyloid and tau burden compared with resilient and vulnerable groups. As expected, vulnerable participants had greater clinical impairment than either canonical or resilient groups. Markers of copathology also differed between tau-clinical mismatch groups. For the tau-PET model, vulnerable participants showed marginally lower TDP-43 MRI signature ERC/PHC ratio (β = −0.23; 95% CI, −0.51 to 0.04; *t*_329_ = −1.66; *P* = .10) and significantly higher rate of α-synuclein positivity (α-synuclein+) (odds ratio [OR], 3.08; 95% CI, 1.52-6.26; *P* = .002) compared with the canonical group (Figure 1B-C). The p-tau217 model showed similar results with a significantly lower TDP-43 MRI signature ERC/PHC ratio (β = −0.28; 95% CI, −0.53 to −0.02; *t*_448_ = −2.15; *P* = .03) and a significantly higher rate of α-synuclein+ (OR, 1.86; 95% CI, 1.04-3.33; *P* = .04) compared with the canonical group (Figure 2B-C). Finally, vulnerable participants in both the tau-PET and p-tau217 models had lower thickness in anterior MTL, temporopolar, and fronto-opercular regions (Figure 1D-E, Figure 2D-E, and eFigures 4 and 5 in Supplement 1).

**Table.** Group Demographics and Summary Measures

Demographic	ADNI tau-PET mismatch, No. (%)			ADNI p-tau217 mismatch, No. (%)			Penn-ADRC mismatch, No. (%)		
	Can (n = 203)	Res (n = 90)	Vuln (n = 72)	Can (n = 299)	Res (n = 124)	Vuln (n = 101)	Can (n = 182)	Res (n = 27)	Vuln (n = 35)
Age, mean (SD), y	73.6 (7.22)	78.5 (7.97)	76.5 (8.27)	75.6 (7.63)	79.5 (7.87)	78.5 (7.91)	73.3 (6.89)	75.2 (6.95)	74.6 (5.98)
Sex									
Female	115 (56.7)	42 (46.7)	35 (48.6)	162 (54.2)	49 (39.5)	45 (44.6)	111 (61)	12 (44.4)	16 (45.7)
Male	88 (43.3)	48 (53.3)	37 (51.4)	137 (45.8)	75 (60.5)	56 (55.4)	71 (39)	15 (55.6)	19 (54.3)
Education, mean (SD), y	16.6 (2.24)	15.6 (2.47)	15.6 (2.58)	16.5 (2.38)	15.6 (2.83)	15.8 (2.6)	16.4 (2.57)	14.9 (2.89)	16.3 (2.92)
Race									
Black	23 (11.3)	7 (7.78)	2 (2.78)	26 (8.70)	8 (6.50)	1 (0.99)	47 (25.8)	11 (40.7)	9 (25.7)
White	172 (84.7)	81 (90.0)	66 (91.7)	262 (87.6)	113 (91.1)	95 (94.1)	129 (70.9)	16 (59.3)	26 (74.3)
Other^a^	8 (3.94)	2 (2.22)	4 (5.56)	11 (3.68)	3 (1.60)	5 (4.95)	6 (3.30)	0	0
Diagnosis									
CU	93 (45.8)	52 (57.8)	0	123 (41.1)	61 (49.2)	0	84 (46.2)	17 (63.0)	0
MCI	89 (43.8)	33 (36.6)	18 (25)	117 (39.1)	52 (41.9)	3 (3)	64 (35.2)	9 (33.3)	6 (17.1)
Dem	21 (10.3)	5 (5.6)	54 (75)	59 (19.7)	11 (8.9)	98 (97)	34 (18.7)	1 (3.7)	29 (82.9)
ApoE ε4									
0	66 (39.5)	41 (50.6)	19 (30.2)	108 (41.2)	54 (47)	28 (28.9)	86 (47.3)	18 (66.7)	8 (22.9)
1	81 (48.5)	29 (35.8)	31 (49.2)	127 (48.5)	44 (38.3)	53 (54.6)	72 (39.5)	7 (25.9)	19 (54.2)
2	20 (12)	11 (13.6)	13 (20.6)	27 (10.3)	17 (14.8)	16 (16.5)	24 (13.1)	2 (7.4)	8 (22.9)
MMSE,^b^ mean (SD)	27.9 (2.46)	27.7 (2.29)	23 (4.48)	26.9 (3.58)	27.2 (2.98)	18.7 (6.3)	27.2 (2.88)	27.6 (2.40)	20.6 (5.58)
Centiloids, mean (SD)	68.2 (38.8)	79.7 (37.5)	89.5 (34.6)	70.2 (38.3)	87.2 (36.6)	93.8 (38.1)	NA	NA	NA
Tau-MaX, mean (SD) [participant No.]	7.99 (16.0)	20.1 (23.9)	17.9 (22.3)	11.4 (19.0) [163]	20.1 (25.4) [61]	24.3 (27.1) [34]	NA	NA	NA
p-Tau217, mean (SD), pg/µL [participant No.]	0.39 (0.34) [138]	0.59 (0.43) [65]	0.64 (0.40) [55]	0.37 (0.30)	0.70 (0.43)	0.65 (0.37)	0.41 (0.31)	0.85 (0.54)	0.59 (0.37)
CDR-SB, mean (SD)	0.98 (1.32)	0.56 (0.82)	5.21 (2.69)	1.57 (1.86)	0.83 (1.15)	8.59 (3.31)	1.26 (1.41)	0.57 (1.05)	7.39 (3.45)
αSyn									
Neg	123 (81.5)	50 (74.6)	32 (56.1)	179 (76.5)	75 (82.4)	47 (59.5)	NA	NA	NA
Pos	28 (18.5)	17 (25.4)	25 (43.9)	55 (23.5)	16 (17.6)	32 (40.5)	NA	NA	NA
ERC/PHC, mean (SD) [participant No.]	0.96 (0.09) [186]	0.95 (0.09) [82]	0.92 (0.10) [68]	0.94 (0.09) [264]	0.96 (0.08) [108]	0.90 (0.09) [80]	0.98 (0.09) [156]	0.98 (0.07) [22]	0.91 (0.13) [31]

Abbreviations: αSyn, α-synuclein; ADNI, Alzheimer’s Disease Neuroimaging Initiative; ApoE, apolipoprotein E; Can, canonical; CDR-SB, Clinical Dementia Rating Sum of Boxes; CU, cognitively unimpaired; Dem, dementia; ERC/PHC, entorhinal cortex thickness/parahippocampal cortex thickness; MCI, mild cognitive impairment; MMSE, Mini-Mental State Examination; NA, not applicable; Neg, negative; p-tau217, phosphorylated tau 217; Penn-ADRC, Penn Alzheimer Disease Research Center; Pos, positive; Res, resilient, Tau-MaX, region-agnostic assessment of global tau magnitude; Vuln, vulnerable.

^a^ Other includes Asian or multiracial.

^b^ MMSE for Penn-ADRC is cross-walked from the Montreal Cognitive Assessment.

### Longitudinal Clinical Trajectories

**Figure 3.**
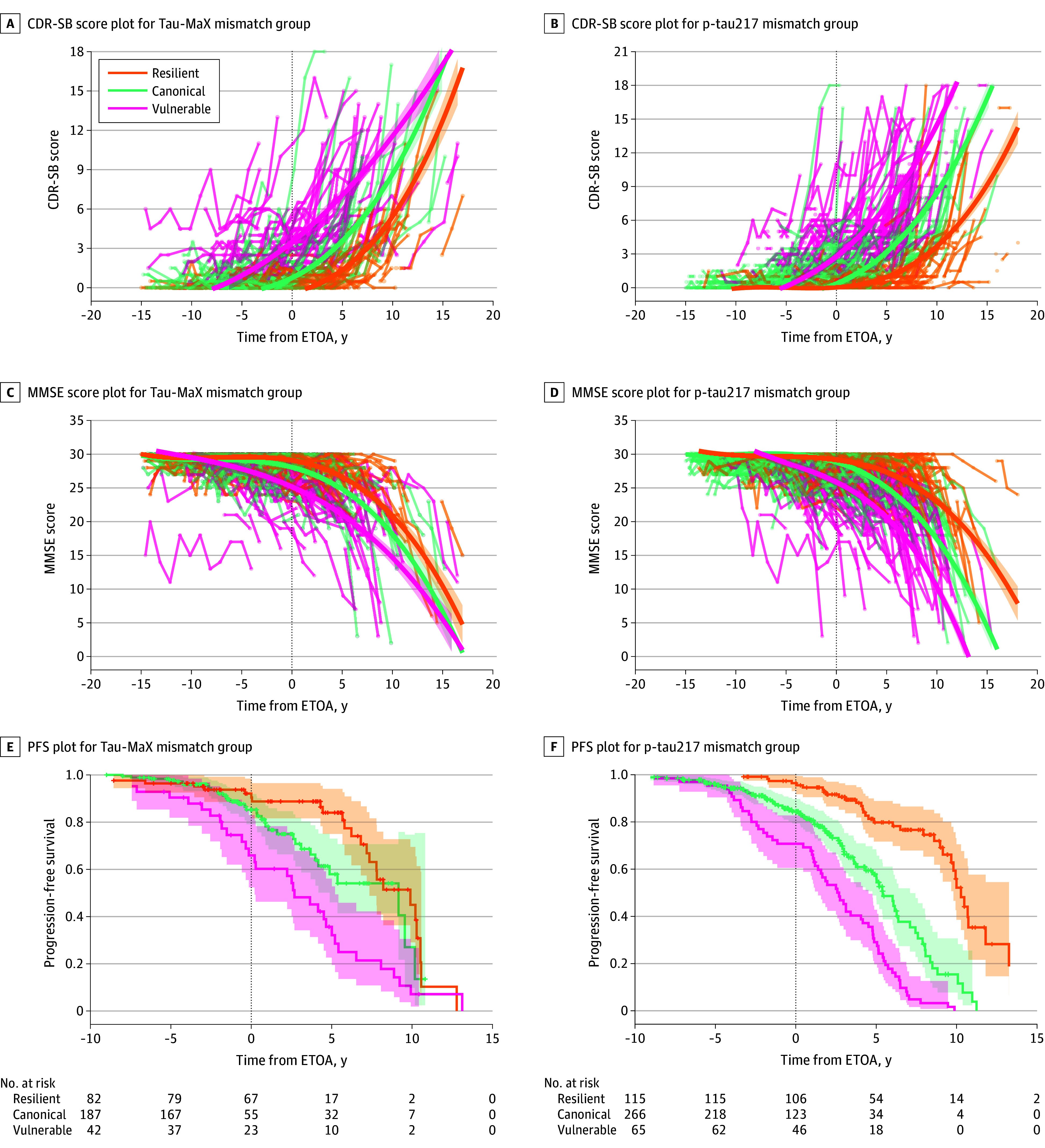
Diverging Longitudinal Trajectories Between Mismatch Groups Longitudinal Clinical Dementia Rating Sum of Boxes (CDR-SB) (A and B), Mini-Mental State Examination (MMSE) (C and D), and progression-free survival (PFS) (E and F) plotted against time from tau positivity for region-agnostic assessment of global tau magnitude (Tau-MaX) and phosphorylated tau 217 (p-tau217) mismatch groups. For CDR-SB and MMSE plots, each point represents individual time points with thin lines connecting time points from the same participant. Thick lines represent the b-spline best fit for each mismatch group (color) with ribbon showing 95% CI of fit. For the PFS plots, time surviving without progression to the next clinical stage (cognitively unimpaired to mild cognitive impairment [MCI] or MCI to dementia) are shown by the survival curve for each mismatch group (color) with ribbon showing the 95% CI of fit. Right-censored data are shown by tick marks on the curves, and number remaining at risk are shown for each 5-year interval. ETOA indicates estimated tau onset age.

We next evaluated the diverging clinical trajectories between tau-clinical mismatch groups by comparing change in CDR-SB and MMSE scores against time anchored to estimated tau onset. For the tau-PET model, the vulnerable group showed faster increase in CDR-SB (*F*_3,1537_ = 22.3; *P* < .001) and decrease in MMSE (*F*_3,1478_ = 14.6; *P* < .001), whereas the resilient group showed slower increase in CDR-SB (*F*_3,1529_ = 17.5; *P* < .001) and decrease in MMSE (*F*_3,1471_ = 3.79; *P* = .01) compared with canonical participants (Figure 3A and C). In the 313 participants who did not have dementia at baseline, 94 transitions to the next clinical stage were observed in longitudinal follow-up with the remaining cases right censored. Compared with canonical participants, vulnerable participants had significantly higher risk for progression to the next stage (hazard ratio [HR], 1.99; 95% CI, 1.22-3.26; *P* = .006), whereas resilient participants had a marginally lower risk of progression (HR, 0.64; 95% CI, 0.38-1.08; *P* = .09) (Figure 3E). Similar results were found for the p-tau217 model, with faster increase in CDR-SB and decrease in MMSE in vulnerable participants (*F*_3,2689 _>48.8; *P* < .001) and the reverse pattern in resilient participants (*F*_3,2635 _>43.2; *P* < .001) for CDR-SB and MMSE compared with the canonical group (Figure 3B and D). In the 449 participants who did not have dementia at baseline, 177 transitions to the next clinical stage were observed in longitudinal follow-up with the remaining cases right censored. Compared with canonical participants, vulnerable participants had significantly higher risk for progression to the next stage (HR, 2.21; 95% CI, 1.58-3.09; *P* < .001), whereas resilient participants had a significantly lower risk of progression (HR, 0.26; 95% CI, 0.17-0.40; *P* < .001) (Figure 3F).

### Replication and Application in Independent Datasets

**Figure 4.**
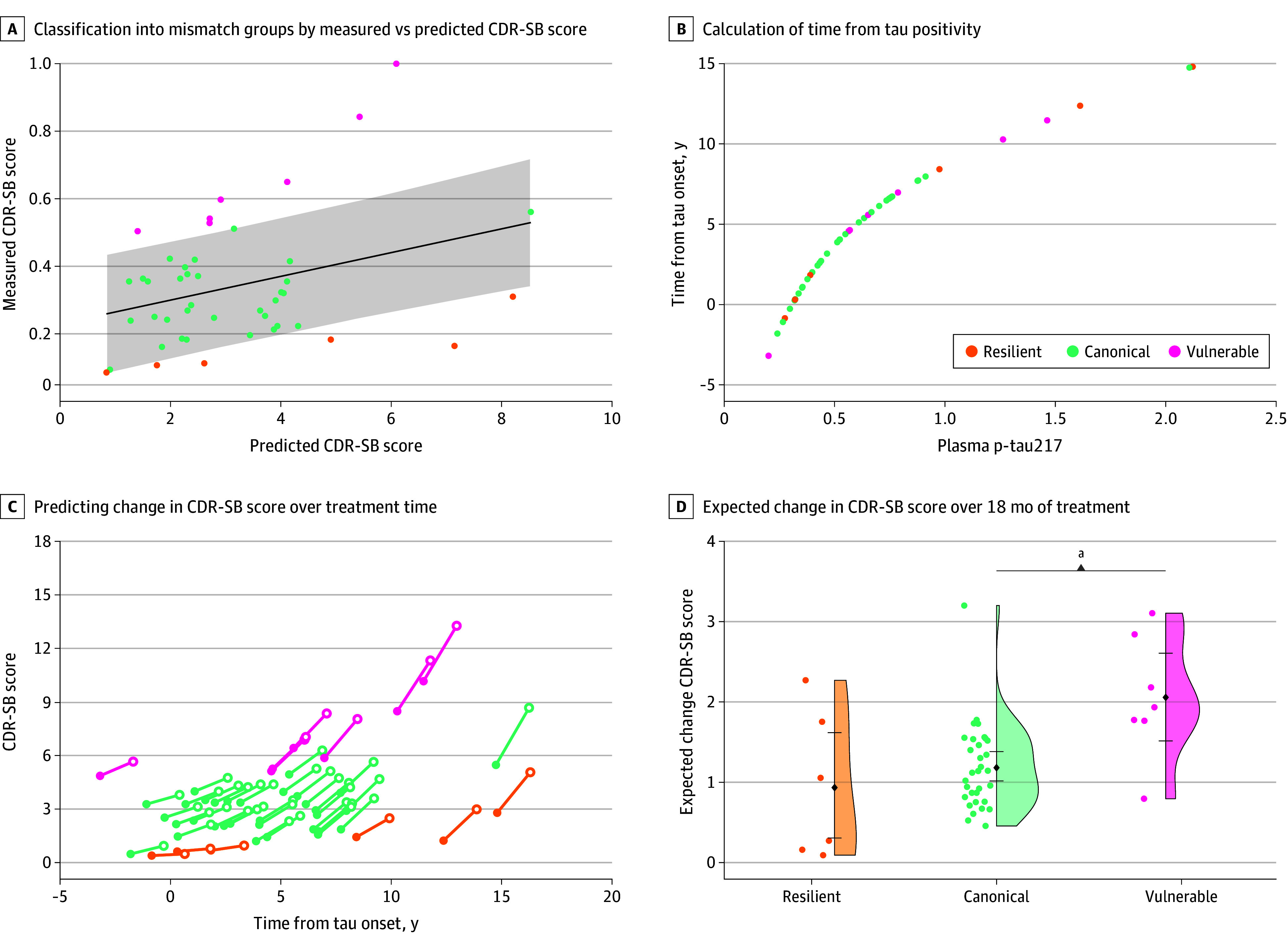
Application of Mismatch Models to Penn Antiamyloid Therapy Monitoring (ATM) Cohort A, Classification of individuals into mismatch groups (color) by comparison of measured Clinical Dementia Rating Sum of Boxes (CDR-SB) against CDR-SB predicted by Alzheimer’s Disease Neuroimaging Initiative (ADNI) mismatch model (β = 0.33; 95% CI, 0.04-0.62; *t* = 2.3; *P* = .03). Gray ribbon represents the threshold of standardized residual (SR) >0.6 from the ADNI model. B, Calculation of time from tau-positivity using phosphorylated tau 217 (p-tau217). C, Predicting change in CDR-SB over treatment time using ADNI mismatch models. Closed circles are the actual CDR-SB measured at baseline, and open circles are predicted CDR-SB after 18 months using longitudinal models generated from ADNI with color indicating mismatch group. D, Expected change in CDR-SB over 18 months of treatment by mismatch group shows significant differences based on mismatch classification. ^a^*P* < .05.

A total of 244 individuals (mean [SD] age, 73.7 [6.8] years; 139 female [56.9%]; 105 male [43.1%]; 1 Asian [0.4%]; 57 Black [23.4%]; 5 multiracial [2.1%]; 171 White [70.1%]) were selected from the 248 Aβ+ individuals in the Penn-ADRC cohort and used to generate tau-clinical mismatch models. We replicated the plasma tau-clinical mismatch model findings in 244 Aβ+ individuals from the Penn-ADRC cohort with 87.7% agreement between mismatch classification using the ADNI model compared with repeating model generation in the Penn-ADRC cohort (eFigure 3A and eTable 2 in Supplement 1). We found similar group differences as were seen in ADNI with lower ERC/PHC ratio (β = −0.57; 95% CI, −0.95 to −0.18; *t*_202_ = −2.91; *P* = .004), lower thickness in anterior MTL and temporopolar regions (eFigure 3B in Supplement 1), and faster clinical progression (eFigure 3C in Supplement 1) in vulnerable compared with canonical groups. Finally, we applied the plasma tau-clinical mismatch model to baseline data from the Penn-ATM cohort, which is a clinical cohort of individuals receiving ATT at the Penn Memory Center. Mismatch classification identified 71% of individuals as canonical, 13% as resilient, and 16% as vulnerable (Figure 4A). We then applied the longitudinal cognitive trajectory models described previously to predict the average expected change in CDR-SB over an 18-month treatment period, illustrating that the expected change over this period differs depending on tau burden and mismatch classification (Figure 4B-D).

## Discussion

In this empiric evaluation of tau-clinical mismatch, we found that individuals classified as cognitively vulnerable appeared to exhibit higher levels of copathology and more rapid clinical decline even after accounting for differences in the level of AD pathology. Moreover, we found that using a more clinically accessible biomarker, plasma p-tau217, as the measure of tau burden in the tau-clinical mismatch model produced nearly identical results as models using tau-PET to measure tau burden. We replicated the primary results from the plasma tau-clinical mismatch model in an independent dataset and illustrated the potential value of these models in a cohort receiving ATT. These models provide clinically accessible tools for assessing the degree to which clinical impairment is driven by AD pathology, the potential influence of copathology and cognitive reserve/resilience, and likely clinical trajectory in the absence of treatment.

Our findings further support the value of the tau-clinical mismatch framework laid out in the 2024 Alzheimer’s Association workgroup diagnostic and staging guidelines.^6^ Similar to a prior study^7^ that used tau-PET to operationalize AD staging, we demonstrated that individuals with greater clinical impairment than expected for a given level of tau pathology (vulnerable) had higher levels of copathology based on direct biomarkers of α-synuclein and indirect biomarkers of TDP-43. In addition, our results suggest that the tau-clinical mismatch framework not only reflected cross-sectional vulnerability or resilience but rather identified individuals on differing longitudinal clinical trajectories. This builds on prior literature demonstrating accelerated clinical progression in those with copathology,^4,25^ as well as studies examining tau-neurodegeneration mismatch.^26,27,28^ However, the tau-clinical mismatch framework likely incorporates differential susceptibility to AD pathology and/or cognitive reserve, which may not be captured by approaches focusing only on copathology or markers of neurodegeneration but likely play an important role in clinical trajectory.^29,30^ Nevertheless, the role of non-AD pathology in clinical progression is suggested by the fact that 33% to 37% of vulnerable individuals already showed cognitive impairment by the time of tau positivity, whereas canonical participants did not reach a similar level until approximately 5 years after tau positivity, a time frame consistent to those previously published in individuals positive for tau-PET.^10^ On the other hand, resilient individuals did not show similar rates of cognitive impairment until more than 10 years after tau positivity, which likely reflects a combination of brain reserve or resilience.^26,30^

The replication of our findings using p-tau217 in place of burden on tau-PET significantly increases the clinical applicability of these models. In contrast to tau-PET, which is primarily confined to research settings, plasma p-tau217 is clinically available and highly accessible. Although its primary clinical use currently is for distinguishing Aβ+/− individuals (sometimes in the form a p-tau217/Aβ42 ratio), numerous studies have shown a stronger association between p-tau217 and tau burden compared to amyloid burden.^8,9,11^ This appears particularly true within Aβ+ individuals, where we demonstrate a stronger association between p-tau217 and Tau-MaX and relatively weak association between p-tau217 and Centiloids when accounting for tau burden. This suggests that p-tau217 can be used as a marker of Aβ positivity but then transitions to a marker of primarily tau burden once individuals are Aβ+. Thus, this association and the similar performance of PET- and plasma-based tau-clinical mismatch models provide further evidence that p-tau217 may be able to provide analogous information about overall tau burden as tau-PET with much greater clinical accessibility. However, future studies may help to further improve plasma-based tau-clinical mismatch models using more specific measures of tau pathology that are completely insensitive to amyloid pathology, but currently, these measures remain in research use only and lack robust, fully automated implementations that could make them clinically feasible.

To illustrate the generalizability and clinical use of the tau-clinical mismatch model, we applied them to an independent dataset and clinical cohort receiving ATT. The strong overlap in tau-clinical mismatch classification when applying the ADNI-generated model compared with refitting the model within the Penn-ADRC dataset, as well as the similar differences between tau-clinical mismatch groups in this independent dataset, suggest strong evidence for the generalizability of these models. Use of these tau-clinical mismatch models could be particularly useful in the setting of ATT or future disease modifying therapies, as illustrated by application to the Penn-ATM cohort. Using our mismatch model, individuals receiving ATT had considerable heterogeneity in expected change in CDR-SB over the 18-month treatment period, thus providing more individualized null models that could serve as a means to measure treatment response in individual patients.^10,31^

### Limitations

A main limitation of these cohorts is that both are volunteer-based cohorts focused on studying Alzheimer disease with predominantly highly educated individuals. Of note, the Penn-ADRC cohort has greater racial representation of the US population compared with ADNI, thus providing evidence that these models may generalize to a broader population. An additional limitation is the lack of longitudinal cognitive assessment in the ATM cohort to validate these models for treatment response, but future studies will be able to investigate this application further. Finally, we do not have autopsy-confirmed pathology, which would provide greater evidence that the structural changes observed in the vulnerable group reflect underlying TDP-43 pathology. Future work in autopsy cohorts with antemortem biomarkers and cognitive assessment should investigate this further.

## Conclusions

In this cohort study, we developed clinically applicable tau-clinical mismatch models that identified individuals more likely to have copathology or cognitive reserve/resilience, predict longitudinal disease progression, and, potentially, could be used to better assess treatment response to ATT in real-world settings.

## Notes

Supplement 1.
eMethods.

eResults.
**eFigure 1.** Cohort Selection Flow Diagram **eFigure 2.** Association Between Tau-Max and p-Tau217 **eFigure 3.** Comparison of Tau-Clinical Mismatch Groups in Penn-ADRC **eFigure 4.** MTL ROI analyses in Tau-Clinical Mismatch Groups **eFigure 5.** Association Between Brain Structure and Continuous Tau-Clinical Mismatch Metric **eTable 1.** Agreement Between Tau-PET and p-Tau217 Mismatch Classification **eTable 2.** Agreement Between Application and Replication Mismatch Classification in Penn-ADRC

Supplement 2. **Nonauthor Collaborators.** Alzheimer’s Disease Neuroimaging Initiative.

Supplement 3.
Data Sharing Statement.
