# Supplementary material for: Evaluation of Copathology and Clinical Trajectories in Individuals With Tau-Clinical Mismatch
Source: JAMA Neurol. 2025 Dec 15;83(2):126–36. doi: 10.1001/jamaneurol.2025.4974 (PMC12706664; doi:10.1001/jamaneurol.2025.4974)
Supplement: Supplement 1. — eMethods. eResults. eFigure 1. Cohort Selection Flow Diagram eFigure 2. Association Between Tau-Max and p-Tau217 eFigure 3. Comparison of Tau-Clinical Mismatch Groups in Penn-ADRC eFigure 4. MTL ROI analyses in Tau-Clinical Mismatch Groups eFigure 5. Association Between Brain Structure and Continuous Tau-Clinical Mismatch Metric eTable 1. Agreement Between Tau-PET and p-Tau217 Mismatch Classification eTable 2. Agreement Between Application and Replication Mismatch Classification in Penn-ADRC [file jamaneurol-e254974-s001.pdf]

Supplementary Online Content

Brown CA, Mundada NS, Cousins KAQ, et al; Alzheimer's Disease Neuroimaging Initiative. Evaluation of copathology and clinical trajectories in individuals with tau-clinical mismatch. *JAMA Neurol*. Published online December 15, 2025.
doi:10.1001/jamaneurol.2025.4974

eMethods.

eResults.

eFigure 1. Cohort Selection Flow Diagram

eFigure 2. Association Between Tau-Max and p-Tau217

eFigure 3. Comparison of Tau-Clinical Mismatch Groups in Penn-ADRC

eFigure 4. MTL ROI analyses in Tau-Clinical Mismatch Groups

eFigure 5. Association Between Brain Structure and Continuous Tau-Clinical Mismatch Metric

eTable 1. Agreement Between Tau-PET and p-Tau217 Mismatch Classification

eTable 2. Agreement Between Application and Replication Mismatch Classification in Penn-ADRC

This supplementary material has been provided by the authors to give readers additional information about their work.

© 2025 Brown CA et al. *JAMA Neurology*.

eMethods.

ADNI Cohort: The ADNI was launched in 2004 as a public-private partnership, led by Principal Investigator Michael W. Weiner, MD. The primary goal of ADNI has been to test whether serial magnetic resonance imaging (MRI), positron emission tomography (PET), other biological markers, and clinical and neuropsychological assessment can be combined to measure the progression of mild cognitive impairment (MCI) and early Alzheimer's disease (AD). For up-to-date information, see www.adni-info.org. All Aβ+ participants (defined by Amyloid PET using ADNI protocols) with at least one Tau-PET or Fujirebio Lumipulse p-tau_{217} and at least one CDR-SB score were included (see eFigure 1).

Penn ADRC: The Penn-ADRC dataset is an ongoing longitudinal observational study with annual evaluation using the Uniform Data Set and consensus diagnosis (REF). Participants were initially selected based on availability of plasma p-tau_{217} and $A\beta_{42}$ measured on Fujirebio Lumipulse platform with a Aβ+ cutoff of p-tau_{217}/$A\beta_{42}$ > 0.0055 selected based on previous data (REF). Aβ+ participants who also had at least one available CDR-SB score were included in analyses (see eFigure 1).

Penn ATM: The Penn-ATM dataset is an ongoing longitudinal observational study of individuals with MCI or Dementia due to AD receiving ATT at the University of Pennsylvania who provide their clinical data and assessments for research us, as well as provide plasma samples collected at infusion visits every 4 weeks. Participants were selected based on availability of p-tau_{217} and clinical assessment with MMSE and DSRS all collected prior to starting ATT.

PET Acquisition and Processing: Tau-PET was acquired using six 5-minute frames from 75-105 minutes after injection of ^{18}F-flortaucipir. Processed ^{18}F-flortaucipir PET images with uniform 6mm full-width-at-half-maximum resolution were downloaded from the ADNI archive ("Coreg, Avg, Std Img and Vox Size, Uniform Resolution"). All PET data were registered to T1-weighted

© 2025 Brown CA et al. *JAMA Neurology*.

images using ANTs rigid-body registration. T1-weighted images were processed as described below; an inferior cerebellar reference region was used to generate SUVR maps and mean SUVR was extracted from cortical regions after partial volume correction. Regional SUVR was used to calculate Global Tau-MaX as previously described (REF). Briefly, SUVR is converted to tau pathology index (TPI) using previously developed Gaussian-mixture models, and then thresholded to include only regions aboe a tau positivity threshold of 2 standard deviations aboe the non-pathologic distribution. TPI in tau+ regions are then multiplied by relative region size and summed to generate Global Tau-MaX, a global measure of tau burden that accounts for both magnitude and extent of disease pathology.

MRI Acquisition and Processing: For ADNI, participants were imaged using 3T MRI scanners to collect T1-weighted structural images. For details of specific protocols, see www.adni-info.org. For Penn-ADRC, T1-weighted images were acquired using a magnetization-prepared gradient-echo (MPRAGE) sequence [repetition time (TR) = 2400 ms, echo time (TE) = 2.24 ms, inversion time (TI) = 1060 ms, flip angle = 8°, 208 axial slices, slice thickness = 0.8 mm, field of view = 240 × 256; in plane resolution = 0.8 mm × 0.8 mm] for a subset of 210 participants. For both ADNI and ABC, images were pre-processed as previously described{Citation}. Briefly, images were bias-corrected and skull-stripped using ANTs prior to cerebellar, cortical, and subcortical parcellation using multi-atlas segmentation with Joint Label fusion with the MICCAI 2012 BrainColor parcellation (REF). ANTs cortical thickness was used to extract the mean cortical thickness from all ROIs (REF).

In addition, MTL segmentation was performed using the T1-ASHS and CRASHS pipeline, which divides the MTL into anterior hippocampus (aH), posterior hippocampus (pH), amygdala, entorhinal cortex (ERC), Brodmann Area (BA) 35, BA36, and parahippocampal cortex (PHC), for ROI and surface-based analyses, respectively[1–4]. For ROI analysis, volume was calculated for aH, pH, and amygdala, while median thickness was extracted for cortical

© 2025 Brown CA et al. *JAMA Neurology*.

MTL regions. For MTL surface-based analyses, pointwise thickness analyses were performed using the mesh_glm tool from the CM-Rep Python package (github.com/pyushkevich/cmrep). General linear model testing was conducted at each vertex of the MTL thickness maps and permutation testing (n = 10000) using threshold-free cluster enhancement (TFCE) to compute family-wise error (FWE) rate-corrected p-values at each vertex.

Plasma Collection and Analysis: Samples were collected as previously described in ADNI and Penn-ADRC (REF). For ADNI, samples were analyzed on a Fujirebio Lumipulse G1200 analyzer at the University of Pennsylvania ADNI Biomarker Core Laboratory (n = 363) or University of Indiana (n = 162). For ADNI, p-tau_{217} concentration from the most recently collected sample was used. For the Penn-ADRC and Penn-ATM, all samples were analyzed on a Fujirebio Lumipulse G1200 analyzer at the University of Pennsylvania ADNI Biomarker Core Laboratory. For Penn-ADRC, p-tau_{217} and $A\beta_{42}$ from the most recently collected sample were used. For Penn-ATM, p-tau_{217} was measured in the sample collected at baseline (within 4 weeks of starting ATT). A cutoff of p-$tau_{217}/A\beta_{42} > 0.0055$ was selected for amyloid positivity based on prior work demonstrating this represents a 95% sensitivity threshold, thus eliminating nearly all individuals who would be negative on PET[5]. Given the goal of the current study, including as many individuals who are Aβ+ was preferred, rather than using a 95% specificity threshold that might exclude a significant number of Aβ+ individuals.

Sampled-Iterative Local Approximation (SILA): As participants were not followed for the same period of time and had measures of tau obtained at various disease stages, we used SILA to place individuals onto a common biological timeline. We generated longitudinal Tau-MaX and p-tau_{217} trajectories using silaR implementation of SILA in a combined dataset of all Aβ+ individuals in ADNI and Penn-ADRC participants with longitudinal Tau-PET and plasma data available as previously described[6]. Briefly, SILA uses discrete sampling to determine the relationship between rate of change in a biomarker at a given biomarker level and then uses

© 2025 Brown CA et al. *JAMA Neurology*.

robust LOESS smoothing and Euler's method to integrate data into a single biomarker trajectory curve[7]. After defining a cutoff value for biomarker positivity, this curve can be plotted against biomarker positivity time to estimate time from biomarker positivity for a given biomarker value[7].

The Tau+ cutoff for Tau-MaX was based on the 97.5%ile of CU Aβ- participants from our previously described dataset, resulting in a cutoff of Tau-MaX > 3.31 (REF). We then used Receiver Operating Curve analysis to evaluate the optimal cutoff based on Youden Index for p-tau_{217} to identify those with Tau-MaX > 3.31 in this same dataset, which resulted in a cutoff of p-tau_{217} > 0.3175. We used these cutoffs for SILA models of Tau-MaX and p-tau_{217} trajectories, respectively, and then generated Estimated Tau Onset Age (ETOA) for all participants using the last available observation with 3 years of interpolation and truncation of estimated ages to the ages observed in the original model.

ATM CDR-SB Crosswalk: As CDR-SB was not available in ATM, we used a cross-walk from the DSRS and MMSE based on historical data from all Penn-ADRC participants with all three measures available. This procedure followed a similar procedure as previously described for DSRS to CDR-SB (REF). The resulting formula was CDR-SB = 7.50 + 0.23 × DSRS – 0.26 × MMSE and had R^2 = 0.83.

Statistical Analyses: Mismatch groups were compared cross-sectionally for differences in markers of co-pathology and brain structure, while controlling for age, sex, education and tau burden (Tau-MaX or p-tau_{217}). For a subset of participants with αSyn (see eFigure 1), we used logistic regression to compare mismatch group differences in odds of αSyn positivity. We also compared the ERC/PHC ratio between mismatch groups using linear regression. For both analyses canonical participants were treated as the reference group and post-hoc pairwise t-tests were used to explore all pairwise comparisons. Next, we compared differences in MTL vertex-wise thickness between mismatch groups using CRASHS as described above with TFCE to compute FWE rate-corrected p-values at each vertex and $p_{FWE} < .05$ considered significant.

© 2025 Brown CA et al. *JAMA Neurology*.

Finally, we evaluated mismatch group differences across cortical ROIs from the whole brain using linear regression with FDR-correction for multiple comparisons and $p_{FDR} < .05$ considered significant. For longitudinal linear-mixed model analyses, we used linear hypothesis comparison testing to evaluate differences in time × mismatch group interaction for all three b-splines of time simultaneously to determine significance.

For the Penn-ADRC dataset, the same analyses as described above were performed with TFCE FWE correction for MTL vertex-wise thickness comparisons and FDR-correction for whole brain cortical ROI analyses. For the Penn-ATM dataset, we classified individuals into mismatch groups using the ADNI p-tau_{217} model. We then used the SILA model described above to determine ETOA. Finally, we used the longitudinal ADNI CDR-SB linear-mixed model to predict change in CDR-SB after 18-months based on ETOA at baseline and mismatch group (along with age, sex, education, and baseline p-tau_{217}). We then compared the predicted change in CDR-SB over an 18-month period based on mismatch group using pairwise t-tests.

© 2025 Brown CA et al. *JAMA Neurology*.

eResults.

Comparison between Tau-MaX and p-tau$_{217}$ for evaluating Tau Burden

In a subset of 258 Aβ+ participants in ADNI with both Tau-PET and p-tau$_{217}$ available, we evaluated the association between p-tau$_{217}$ and Tau-MaX as well as with Centiloids (eFigure 2). There was a strong positive association between Tau-MaX and p-tau$_{217}$ (β = 0.71 [0.63, 0.80], $t(256) = 16.3$, $p < .001$), while p-tau$_{217}$ and Centiloids had a moderate positive association (β = 0.46 [0.35, 0.57], $t(256) = 8.24$, $p < .001$). When included in the same model, Tau-MaX had a strong positive association with p-tau$_{217}$ (β = 0.63 [0.54, 0.72], $t(255) = 13.6$, $p < .001$), while Centiloids had a weak positive association with p-tau$_{217}$ (β = 0.20 [0.11, 0.29], $t(255) = 4.33$, $p < .001$). Overall, these findings support p-tau$_{217}$ as being a closer approximate of tau burden within Aβ+ individuals than of amyloid burden, and lend support to its use for evaluating tau-clinical mismatch. We also evaluated the similarity between mismatch classifications when using Tau-PET and p-tau$_{217}$ in this group with both measures available (eTable 1), which also shows high agreement between methods.

Mismatch Classification and Group Comparisons in Penn-ADRC

There was high agreement between mismatch classification when applying the ADNI model and regenerating the model in Penn-ADRC (eTable 2). Cross-sectional brain structure differences and longitudinal cognitive trajectories are shown in eFigure 3 with similar findings to the ADNI dataset.

Application of Tau-Clinical Mismatch Classifications to Penn-ATM Cohort

When using Tau-Clinical Mismatch classification, estimation of tau onset age, and applying the longitudinal clinical models to estimate predicted change in CDR-SB over 18-months, we found significant differences in expected progression by mismatch group, with greater change in CDR-

© 2025 Brown CA et al. *JAMA Neurology*.

SB predicted in the vulnerable group ($t(6.7) = -4.57$, $p = .003$) and smaller change predicted in the resilient group ($t(6.3) = 2.47$, $p = .046$).

ROI Anlayses of MTL structure in ADNI and Penn-ADRC datasets

For the ADNI tau-PET dataset, vulnerable participants showed lower aH pH, and amygdala volume ($p \leq .019$), as well as lower ERC, BA35, and PHC thickness ($p \leq .026$) compared to canonical participants (eFigure 4A). The resilient group showed the opposite pattern with higher aH, pH, and amygdala volume ($p \leq .008$), as well as greater thickness in ERC, BA35, and PHC ($p < .001$) compared to the canonical group (eFigure 4A). For the ADNI p-tau_{217} dataset, vulnerable participants showed lower volume or thickness across all MTL regions ($p \leq .013$), while resilient participants showed higher volume or thickness across all MTL regions ($p \leq .009$) compared to the canonical group (eFigure 4B). For the ABC dataset, vulnerable participants showed lower volume or thickness across all MTL regions ($p \leq .007$), while the resilient group did not differ compared to the canonical group in any MTL region (eFigure 4C).

Mismatch as a Continuous Measure

We repeated analyses treating mismatch as a continuous measure and found a negative association between mismatch residual (more positive value = more vulnerable) and volume or thickness in all MTL regions for both Tau-MaX ($p < .001$) and p-tau_{217} ($p < .001$) defined mismatch after controlling for Tau-MaX (eFigure 3). We also found a negative association between mismatch residual and ERC/PHC ratio for both Tau-MaX ($p < .001$) and p-tau_{217} ($p < .001$) defined mismatch (eFigure 3), as well as higher risk of αSyn+ as Tau-MaX (OR = 1.32 [1.03, 1.70], $p = .027$) or p-tau_{217} (OR = 1.41 [1.15, 1.73], $p = .001$) mismatch residual increased.

Use of Tau-MaX as a Control for Global Tau-Burden in ADNI PET-Plasma Cohort

© 2025 Brown CA et al. *JAMA Neurology*.

We repeated comparisons between plasma-defined mismatch groups in the ADNI cohort that had both Tau-PET and p-tau_{217} available to assess if similar findings were seen when using Tau-MaX as a control for global tau burden when comparing between mismatch groups rather than p-tau_{217}. We found similar findings with the exception of the ERC/PHC ratio no longer being significant in this cohort (which matches its lack of significance in the Tau-PET cohort). In contrast, vulnerable participants still showed significantly higher rates of αSyn+ (OR = 3.39 [1.46, 7.92], p = .004) and faster cognitive decline ($F_{3,1573}$ = 42.2, p < .001) compared to canonical participants, while resilient participants showed slower cognitive decline ($F_{3,1549}$ = 13.5, p < .001).

© 2025 Brown CA et al. *JAMA Neurology*.

eFigure 1. Cohort selection flow diagram

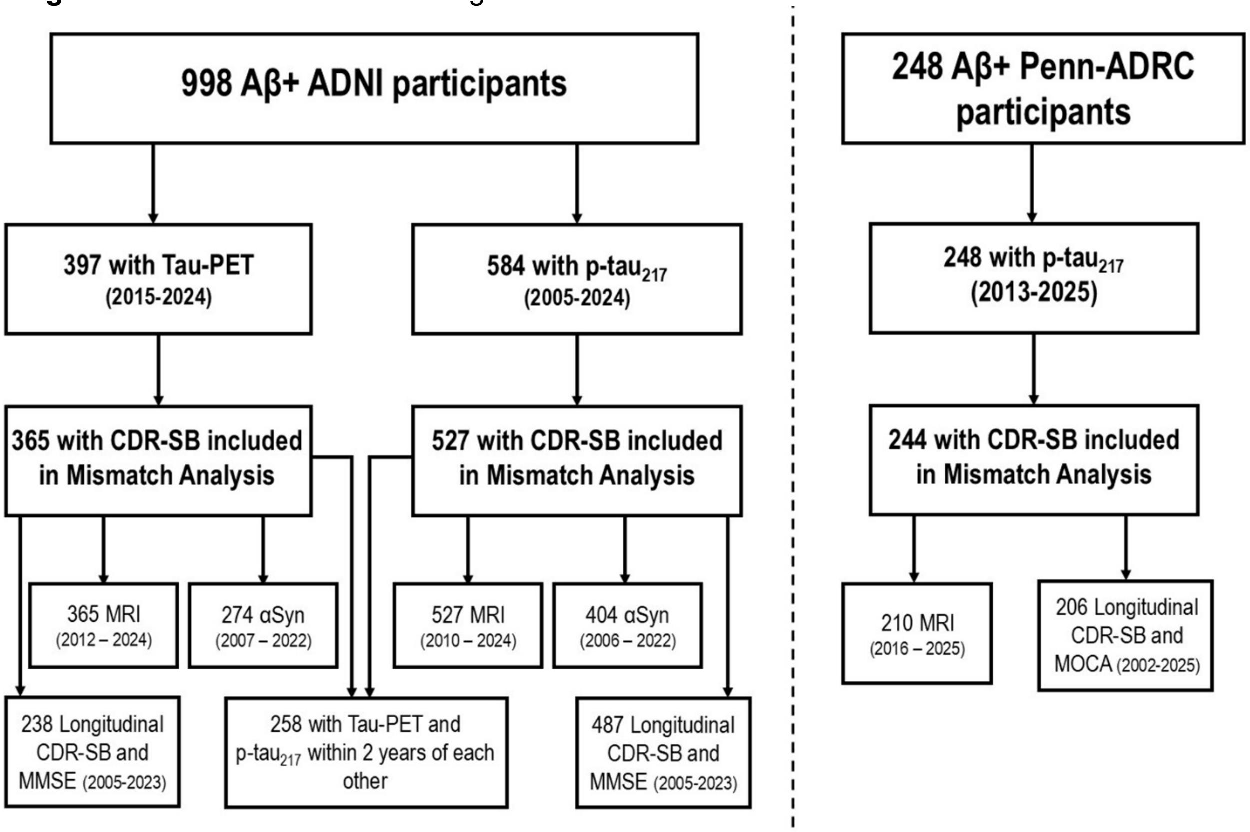

eFigure 1. Cohort selection flow diagram. The method for selecting participants from ADNI (left) and Penn-ADRC (right) is shown. Bolded boxes demonstrate the flow to form the cohorts for tau-clinical mismatch analyses. The remaining boxes show subsets of participants included in relevant analyses. The years data were collected are included for each measure.

© 2025 Brown CA et al. *JAMA Neurology*.

eFigure 2. Association between Tau-MaX and p-tau$_{217}$

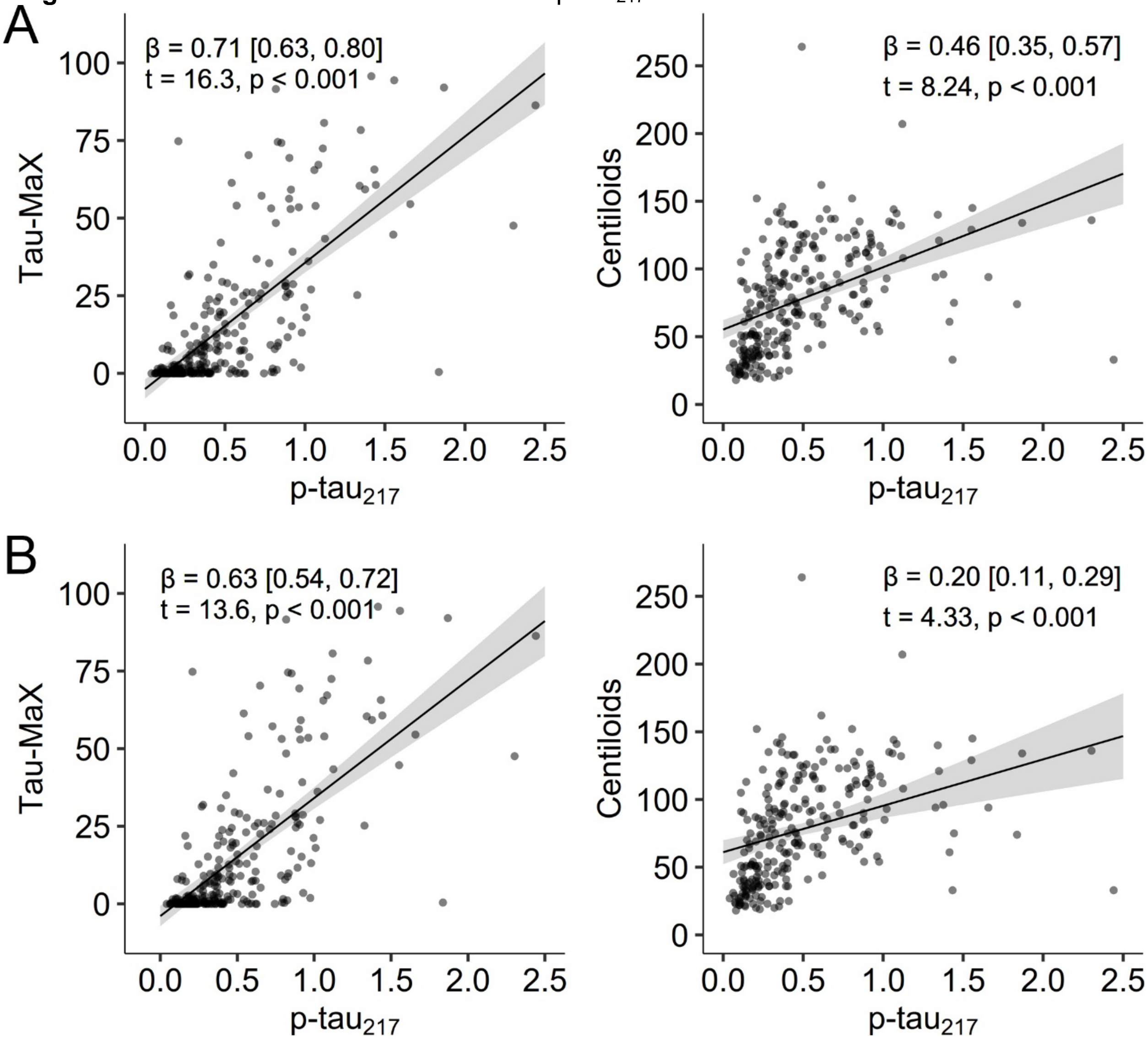

eFigure 2. Association between Tau-MaX and p-tau$_{217}$. A: The univariate relationship of p-tau$_{217}$ with Tau-MaX (left) and Centiloids (right) in the 258 Aβ+ participants with both measures available. **B:** The multivariate association between p-tau$_{217}$ with Tau-MaX (left) and Centiloids (right) when included in the same model. **Both:** The linear best-fit is shown with shading representing the 95% confidence of fit. Standardized β values are shown along with [95% confidence intervals].

© 2025 Brown CA et al. *JAMA Neurology*.

eFigure 3. Comparison of Tau-Clinical Mismatch Groups in Penn-ADRC

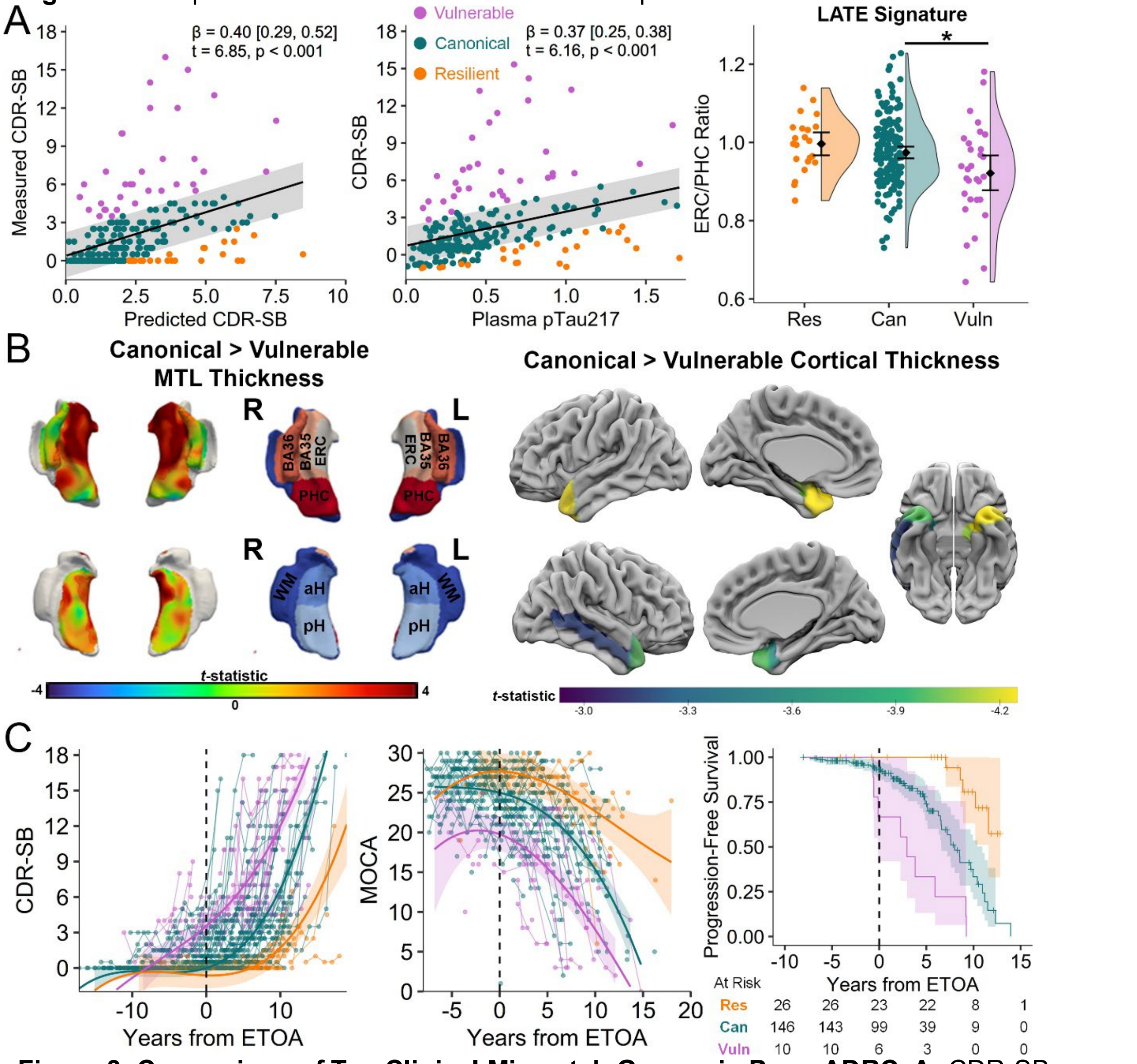

eFigure 3. Comparison of Tau-Clinical Mismatch Groups in Penn-ADRC. A: CDR-SB plotted against ADNI-model predicted CDR-SB (left) and p-tau$_{217}$ within ABC (middle) with ADNI-based mismatch classification shown by color scale for both. Gray shading represents Standardized Residual (SR) = 0.6. Comparison of the LATE MRI signature ERC/PHC thickness ratio between mismatch groups (right), **p* < .05. **B:** Differences in MTL thickness (left) and whole brain ROI cortical thickness (right) between canonical and vulnerable groups with color-scale representing the t-statistic for group comparison. Clusters surviving TFCE $p_{FWE} < .05$ are outlined in black for MTL analyses. Only regions surviving $p_{FDR} < .05$ are shown for whole brain ROI analyses. C: Longitudinal CDR-SB (left) and MOCA (middle) plotted against time from Tau positivity for mismatch groups. Each point represents individual time points with thin lines connecting time points from the same participant. Thick lines represent the b-spline best-fit for each mismatch group (color) with ribbon showing 95% confidence interval of fit. Time surviving without progression to the next clinical stage based on years of tau positivity for mismatch groups is shown on the right. Lines represent the survival curve for each mismatch group (color) with ribbon showing the 95% confidence interval of fit.

© 2025 Brown CA et al. *JAMA Neurology*.

eFigure 4. MTL ROI analyses in Tau-Clinical Mismatch Groups

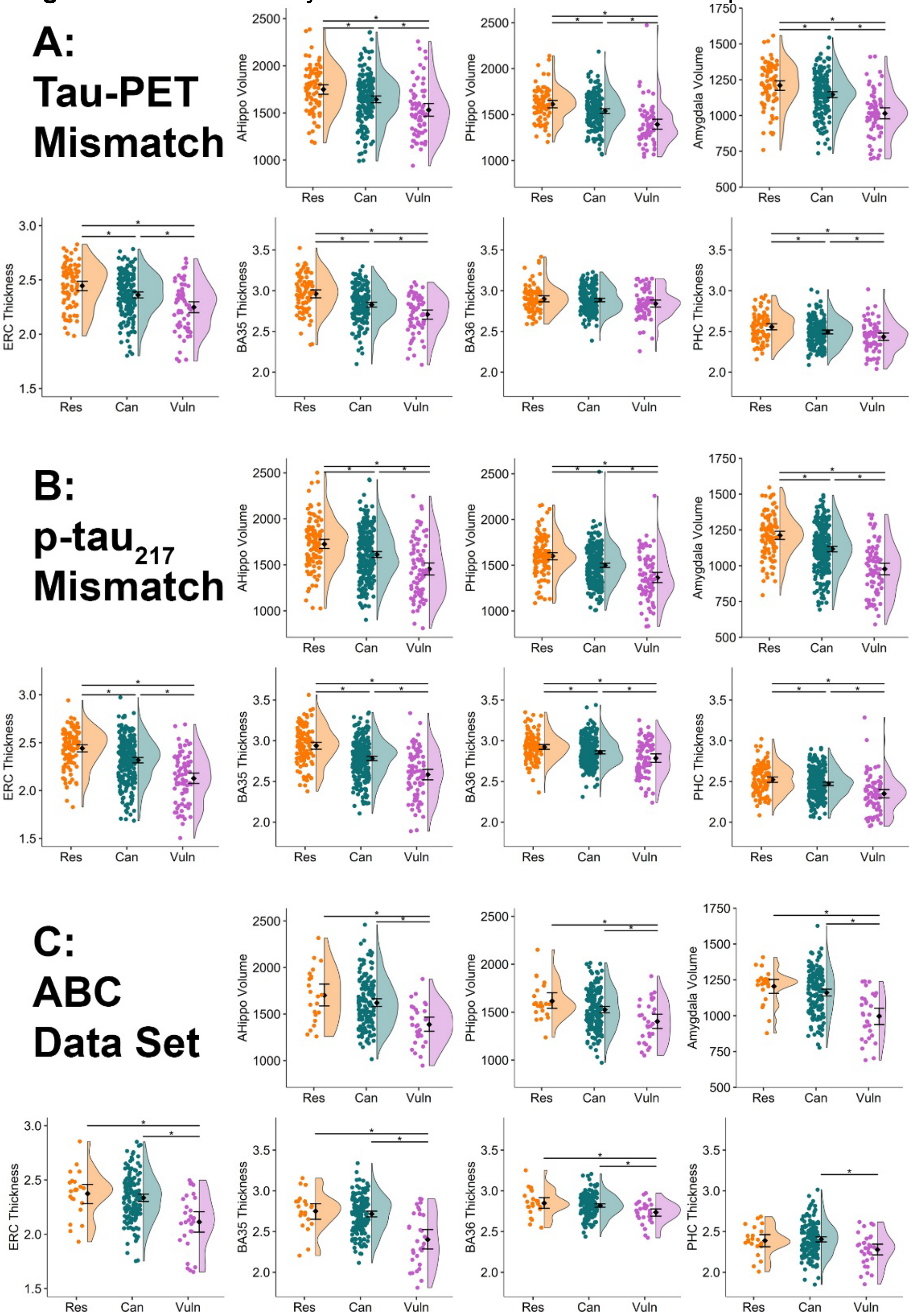

eFigure 4. MTL ROI analyses in Tau-Clinical Mismatch Groups. Group comparisons within MTL regions in the Tau-PET **(A)**, p-tau$_{217}$ **(B)**, and Penn ABC (**C)** dataset are shown. In each panel, Anterior Hippocampus (AHippo), Posterior hippocampus (PHippo), and amygdala volume are shown in the top row and entorhinal cortex (ERC), Brodman Area (BA) 35, BA36, and parahippocampal cortex (PHC) thickness are shown in the bottom row. Each data point represents an individual and significant differences after FDR-correction are shown with bars. *$p_{FDR} < 0.05$.

© 2025 Brown CA et al. *JAMA Neurology*.

eFigure 5. Association between brain structure and continuous Tau-Clinical mismatch metric

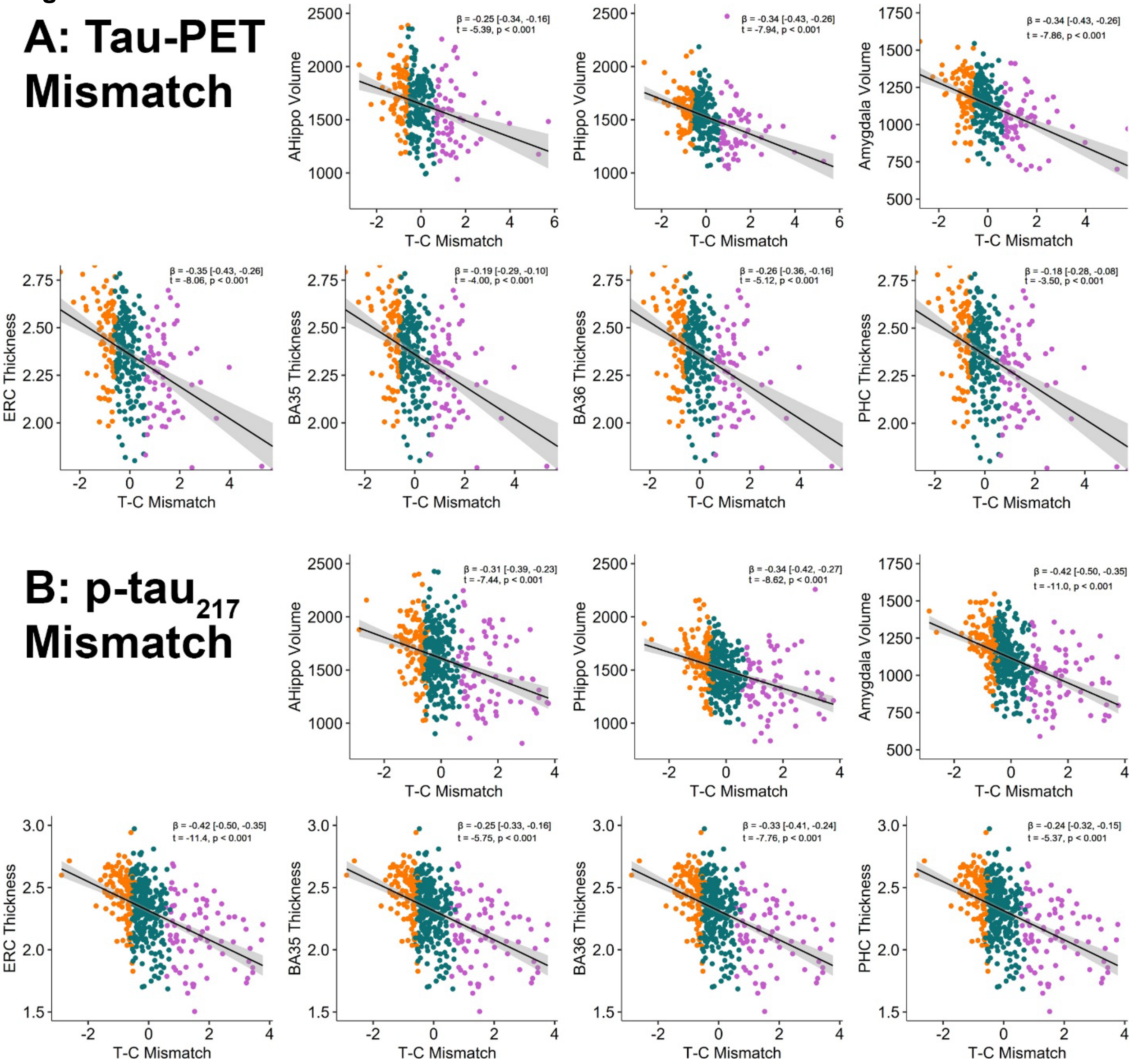

eFigure 5. Association between brain structure and continuous Tau-Clinical mismatch metric. Association between the residual from the Tau-MaX **(A)** or p-tau$_{217}$ **(B)** association with CDR-SB (T-C Mismatch) and MTL structure are shown. Colors of each point represent the mismatch group assignment from group-based analyses. The standardized *β* [95% CI], *t*-test, and *p*-value for each association are shown.

© 2025 Brown CA et al. *JAMA Neurology*.

eTable 1. Agreement between Tau-PET and p-tau_{217} mismatch classification

		Tau-PET Mismatch			Plasma Total
		Resilient	Canonical	Vulnerable	
**Plasma Mismatch**	Resilient	**39 (15.1%)**	21 (8.1%)	1 (0.4%)	61 (23.2%)
	Canonical	26 (10.1%)	**113 (43.8%)**	24 (9.3%)	163 (63.2%)
	Vulnerable	0 (0%)	4 (1.6%)	**30 (11.6%)**	34 (13.2%)
**Tau-PET Total**		65 (25.2%)	138 (51.9%)	55 (21.3%)	258

Mismatch agreement between plasma p-tau_{217} and Tau-PET is shown with n (%) for each group. Cases of agreement are bolded

© 2025 Brown CA et al. *JAMA Neurology*.

eTable 2. Agreement between application and replication mismatch classification in Penn-ADRC

		**ADRC Regeneration**			**Application Total**
		Resilient	Canonical	Vulnerable	
**ADNI Application**	Resilient	**25 (15.1%)**	2 (0.8%)	0 (0%)	27 (15.9%)
	Canonical	24 (9.8%)	**155 (63.5%)**	3 (1.2%)	182 (74.5%)
	Vulnerable	0 (0%)	1 (0.4%)	**34 (11.6%)**	35 (12.0%)
**Regeneration Total**		49 (24.9%)	158 (64.7%)	37 (12.8%)	244

Mismatch agreement between application of the ADNI model to Penn-ADRC versus regeneration of the mismatch model in Penn-ADRC is shown with n (%) for each group. Cases of agreement are bolded

© 2025 Brown CA et al. *JAMA Neurology*.
