# Supplementary material for: Evaluation of Copathology and Clinical Trajectories in Individuals With Tau-Clinical Mismatch
Source: JAMA Neurol. 2025 Dec 15;83(2):126–36. doi: 10.1001/jamaneurol.2025.4974 (PMC12706664; doi:10.1001/jamaneurol.2025.4974)
Supplement: Supplement 2. — Nonauthor Collaborators. Alzheimer’s Disease Neuroimaging Initiative. [file jamaneurol-e254974-s002.pdf]

*First name, last name, and suffix (if applicable) are required and will appear in PubMed.

***Group Name(s):** Alzheimer's Disease Neuroimaging Initiative

***First Name and Middle Initial(s)**	***Last Name**	***Suffix (eg, Jr, III)**	Academic Degrees	Institution	Location (city, state/province, country)	Role or Contribution, eg, chair, principal investigator	Group (if more than 1 Group listed in the byline) and/or Subgroup (eg, Steering Committee)
Olusegun	Adegoke		MSc	University of Southern California			
Kedir	Adem Hussen		MS	University of Southern California			
Paul	Aisen		MD	University of Southern California		ATRI PI	
Adeyinka	Ajayi		MBBS	MPH Mt. Sinai			
Hannatu	Amaza			University of Wisconsin			
Liana G.	Apostolova		MD	Indiana University School of Medicine			
Miriam	Ashford		PhD	Northern California Institute for Research and Education			
Omobolanle	Ayo		MBChB	MPH Mt. Sinai			
Lisa	Barnes		PhD	Rush University			
Laurel	Beckett		PhD	University of California Davis		Core PI	
Marie	Bernard		MD	NIA			
Haley	Bernhardt		BA R. EEG	Washington University St. Louis			
Virginia	Boatwright		BS	University of Southern California			
Bret	Borowski		RTR	Mayo Clinic	Rochester		
Magdalena	Brylska		MS	UPenn School of Medicine			
Neil	Buckholtz		PhD	National Institute on Aging			
Yuliana	Cabrera		BS	University of Southern California			
Nigel J.	Cairns		PhD	MRCPath Washington University St. Louis			
Maria	Carrillo		PhD	Alzheimer’s Association			
Mark	Choe		BS	Northern California Institute for Research and Education			
Taylor	Clanton		MPH	University of Southern California			
Cat	Conti		BA	Northern California Institute for Research and Education			

Supplemental Online Content: Nonauthor Collaborators
*First name, last name, and suffix (if applicable) are required and will appear in PubMed.

***First Name and Middle Initial(s)**	***Last Name**	***Suffix (eg, Jr, III)**	Academic Degrees	Institution	Location (city, state/province, country)	Role or Contribution, eg, chair, principal investigator	Group (if more than 1 Group listed in the byline) and/or Subgroup (eg, Steering Committee)
Hannah	Craft		MPH	Indiana University School of Medicine			
Karen	Crawford		MLIS	University of Southern California			
Sandhitsu	Das		PhD	University of Pennsylvania			
Charles	DeCarli		MD	University of California Davis			
Joseph	Di Benedetto		LMSW	Mt. Sinai			
Adam	Diaz		MS	Northern California Institute for Research and Education			
Michael	Donohue		PhD	University of Southern California			
Erin	Drake			Harvard University			
Claire	Erickson		PhD	University of Pennsylvania			
Kelley	Faber		MS	CCRC NCRAD/Indiana University School of Medicine			
Joel	Felmlee		PhD	Mayo Clinic	Rochester		
Andrea	Fidell		MPH	University of Southern California			
Derek	Flenniken			Northern California Institute for Research and Education			
Evan	Fletcher		PhD	University of California Davis			
Juliet	Fockler			University of California San Francisco			
Arvin	Forghanian-Arani		PhD	Mayo Clinic	Rochester		
Tatiana M.	Foroud		PhD	Indiana University School of Medicine		Dir. NCRAD	
Nick C.	Fox		MD	University College London			
Richard	Frank		MD PhD	General Electric			
Erin	Franklin		MS	Washington University St. Louis			
Matt	Glittenberg			University of Wisconsin			
Hector	González			University of California San Diego			
Robert C.	Green		MD PPH	Harvard University (Chair)			
Joshua	Grill		PhD	University of California Irvine			
Jeff	Gunter		PhD	Mayo Clinic	Rochester		
Vanessa	Guzman		PhD	Mt. Sinai			

Supplemental Online Content: Nonauthor Collaborators
*First name, last name, and suffix (if applicable) are required and will appear in PubMed.

*First Name and Middle Initial(s)	*Last Name	*Suffix (eg, Jr, III)	Academic Degrees	Institution	Location (city, state/province, country)	Role or Contribution, eg, chair, principal investigator	Group (if more than 1 Group listed in the byline) and/or Subgroup (eg, Steering Committee)
Kristin	Harkins			MPH University of Pennsylvania			
Danielle	Harvey		PhD	University of California Davis		Core PI	
Caitie	Hedberg			Mayo Clinic	Rochester		
Lindsey	Hergesheimer		BS	University of Southern California			
Carole	Ho			Denali Therapeutics			
Isabella	Hoang			University of Wisconsin			
John K.	Hsiao		MD	National Institute on Aging			
Clifford R.	Jack	Jr.	MD	Mayo Clinic	Rochester		
Jonathan	Jackson		PhD	Massachusetts General Hospital			
William	Jagust		MD	University of California Berkelely		Core PI	
Neda	Jahanshad		PhD	University of Southern California School of Medicine			
Cecily	Jenkins		PhD	University of Southern California			
Gustavo	Jimenez		MBS	University of Southern California			
Chengshi	Jin		PhD	University of California San Francisco			
Taeho	Jo		PhD	Indiana University School of Medicine			
Zaven	Kachaturian		PhD	Khachaturian Radebaugh & Associates (KRA) Inc			
Rima	Kaddurah-Daouk		PhD	Duke University/AD Metabolomics Consortium			
Kejal	Kantarci		MD	Mayo Clinic	Rochester		
Jason	Karlawish		MD	University of Pennsylvania			
Zaven	Khachaturian		PhD	Prevent Alzheimer's Disease 2020		Chair	
Alexander	Knaack		MS	University of California Davis			
Robert A.	Koeppe		PhD	University of Michigan			
Magdalena	Korecka		PhD	UPenn School of Medicine			
Adrienne	Kormos			Northern California Institute for Research and Education			
Kaori	Kubo Germano		PhD	Fordham University			
Winnie	Kwang		MA	University of California San Francisco			

*First name, last name, and suffix (if applicable) are required and will appear in PubMed.

*First Name and Middle Initial(s)	*Last Name	*Suffix (eg, Jr, III)	Academic Degrees	Institution	Location (city, state/province, country)	Role or Contribution, eg, chair, principal investigator	Group (if more than 1 Group listed in the byline) and/or Subgroup (eg, Steering Committee)
Kaci	Lacy		MPH	CCRP NCRAD/Indiana University School of Medicine			
Susan	Landau		PhD	University of California Berkeley		Core PI	
Emily	Largent		PhD	University of Pennsylvania			
Edward B.	Lee		MD PhD	University of Pennsylvania		Core PI	
Virginia M.Y.	Lee		PhD	MBA UPenn School of Medicine			
Brian	LoPresti			University of Pittsburgh			
Fabiola	Magana			University of Wisconsin			
Payam	Mahboubi		MPH	University of Southern California			
Ian	Malone		PhD	University College London			
Eliezer	Masliah		MD	NIA			
Donna	Masterman		MD	Biogen			
Leonard	Matoush			Mayo Clinic	Rochester		
Melanie J.	Miller		PhD	Northern California Institute for Research and Education			
Susan	Molchan		PhD	National Institute on Aging/National Institutes of Health			
Tom	Montine		MD PhD	University of Washington (Chair)			
John	Moore-Weiss		PhD	Mayo Clinic	Rochester		
John	Morris		MD	Washington University St. Louis			
Scott	Neu		PhD	University of Southern California			
Kwangsik	Nho		PhD	Indiana University School of Medicine		Core PI	
Talia M.	Nir		PhD	University of Southern California School of Medicine			
Rachel	Nosheny		PhD	University of California San Francisco			
Kelly	Nudelman		PhD	NCRAD/Indiana University School of Medicine			
Sheila	Ogwang		MPH	University of Southern California			
Ozioma	Okonkwo		PhD	University of Wisconsin (Core-PI)		Core PI	

Supplemental Online Content: Nonauthor Collaborators
*First name, last name, and suffix (if applicable) are required and will appear in PubMed.

***First Name and Middle Initial(s)**	***Last Name**	***Suffix (eg, Jr, III)**	Academic Degrees	Institution	Location (city, state/province, country)	Role or Contribution, eg, chair, principal investigator	Group (if more than 1 Group listed in the byline) and/or Subgroup (eg, Steering Committee)
Shaniya	Parkins			Mt Sinai			
Richard	Perrin		MD PhD	Washington University St. Louis			
Ronald	Petersen		MD PhD	Mayo Clinic	Rochester	Core PI	
Jeremy	Pizzola		BA	University of Southern California			
Zoë	Potter		BA	CCRP NCRAD/Indiana University School of Medicine			
William	Potter		MD	National Institute of Mental Health			
Gil	Rabinovici			University of California San Francisco			
Michael	Rafii		MD PhD	University of Southern California School of Medicine			
Rema	Raman		PhD	University of Southern California			
Robert	Reid		PhD	Mayo Clinic	Rochester		
Calvin	Reyes			Mayo Clinic	Rochester		
Denise	Reyes			Mayo Clinic	Rochester		
Shannon L.	Risacher		PhD	Indiana University School of Medicine			
Monica	Rivera-Mindt		PhD	Fordham University; Mt. Sinai		Core PI	
Justin	Robison		MS	University of Southern California			
Stephanie	Rossi Chen		BA	Northern California Institute for Research and Education			
Laurie	Ryan		PhD	National Institute on Aging			
Pallavi	Sachdev		PhD	Eisai		Chair 2023-2024	
Naomi	Saito		MS	University of California Davis			
Jennifer	Salazar		MBS	University of Southern California			
Andrew J.	Saykin		PsyD	Indiana University School of Medicine		Core PI	
Christopher	Schwarz		PhD	Mayo Clinic	Rochester		
Mai	Seng Thao			University of Wisconsin			
Matthew	Senjem		MS	Mayo Clinic	Rochester		

Supplemental Online Content: Nonauthor Collaborators
*First name, last name, and suffix (if applicable) are required and will appear in PubMed.

***First Name and Middle Initial(s)**	***Last Name**	***Suffix (eg, Jr, III)**	Academic Degrees	Institution	Location (city, state/province, country)	Role or Contribution, eg, chair, principal investigator	Group (if more than 1 Group listed in the byline) and/or Subgroup (eg, Steering Committee)
Elizabeth	Shaffer		BS	University of Southern California			
Leslie M.	Shaw		PhD	University of Pennsylvania			
Li	Shen		PhD	University of Pennsylvania			
Nina	Silverberg		PhD	NIA			
Stephanie	Smith		BS	University of Southern California			
Peter J.	Snyder		PhD	University of Connecticut			
Joe	Strong		PhD	University of Wisconsin			
Sandra	Talavera		MSW	Fordham University			
Lisa	Taylor-Reinwald		BA HTL	Washington University St. Louis			
Leon	Thal		MD			Past Investigator	
Lisa	Thomas			University of Wisconsin			
Sophia I.	Thomopoulos		BS	University of Southern California School of Medicine			
Paul	Thompson		PhD	UCLA School of Medicine			
Arthur W.	Toga		PhD	University of Southern California		Core PI	
Duygu	Tosun		PhD	University of California San Francisco			
J.Q.	Trojanowki		MD PhD	UPenn School of Medicine		former Core PI	
Diana	Truran Sacrey			Northern California Institute for Research and Education			
Prashanthi	Vemuri		PhD	Mayo Clinic	Rochester		
Victor	Villemagne			University of Pittsburgh			
Sarah	Walter		MSc	University of Southern California			
Yang	Wan		MS	UPenn School of Medicine			
Chad	Ward			Mayo Clinic	Rochester		
Caitlin	Webb		BA	University of Southern California			
Michael	Weiner		MD	University of California San Francisco		PI	
Trinity	Weisensel			University of Wisconsin			

Supplemental Online Content: Nonauthor Collaborators
*First name, last name, and suffix (if applicable) are required and will appear in PubMed.

***First Name and Middle Initial(s)**	***Last Name**	***Suffix (eg, Jr, III)**	Academic Degrees	Institution	Location (city, state/province, country)	Role or Contribution, eg, chair, principal investigator	Group (if more than 1 Group listed in the byline) and/or Subgroup (eg, Steering Committee)
Paul A.	Yushkevich		PhD	University of Pennsylvania			
Caileigh	Zimmerman		MS	University of Southern California			
