# Supplementary material for: Evaluation of Copathology and Clinical Trajectories in Individuals With Tau-Clinical Mismatch
Source: JAMA Neurol. 2025 Dec 15;83(2):126–36. doi: 10.1001/jamaneurol.2025.4974 (PMC12706664; doi:10.1001/jamaneurol.2025.4974)
Supplement: Supplement 3. — Data Sharing Statement. [file jamaneurol-e254974-s003.pdf]

Data Sharing Statement

Brown. Evaluation of Copathology and Clinical Trajectories in Individuals With Tau-Clinical Mismatch. *JAMA Neurol*. Published December 15, 2025. doi:10.1001/jamaneurol.2025.4974

Data
Data available: Yes
Data types: Deidentified participant data, Data dictionary
How to access data: All requests for raw and analyzed data from the Penn ADRC and ATM cohort will be reviewed by the Penn Neurodegenerative Data Sharing Committee (PNDSC) and shared for appropriate uses through a data sharing agreement (https://www.pennbindlab.com/data-sharing). Anonymized data from the Penn ADRC and ATM will be shared upon request to the corresponding author by a qualified academic investigator for the purpose of replicating procedures and results in this article. Data are not publicly available due to privacy protections outlined in the participant informed consent. Documents related to study protocols, informed consent and other documentation can similarly be made available upon request. All ADNI data are shared without embargo through the LONI Image and Data Archive (https://ida.loni.usc.edu/), a secure research data repository. Interested scientists may obtain access to ADNI imaging, clinical, genomic, and biomarker data for the purposes of scientific investigation, teaching, or planning clinical research studies. Access is contingent on adherence to the ADNI Data Use Agreement and the publications' policies (https://adni.loni.usc.edu/data-samples/access-data/).
When available: With publication

Supporting Documents
Document types: Statistical/analytic code
How to access documents: Statistical and analytic code can be obtained by emailing the corresponding author (christopher.brown@pennmedicine.upenn.edu)
When available: With publication

Additional Information
Who can access the data: Deidentified participant data will be made available to researchers whose proposed use of the data has been approved. Statistical and analytic code will be made available to anyone requesting the code.
Types of analyses: Deidentified participant data may be used for the purpose specified in the data request and/or for the purpose of replicating the procedures and results of this study.
Mechanisms of data availability: Data will be made available after approval of a proposal and signed data access agreement.
